# Spatio-temporal mapping of Madagascar’s Malaria Indicator Survey results to assess *Plasmodium falciparum* endemicity trends between 2011 and 2016

**DOI:** 10.1186/s12916-018-1060-4

**Published:** 2018-05-23

**Authors:** Su Yun Kang, Katherine E. Battle, Harry S. Gibson, Arsène Ratsimbasoa, Milijaona Randrianarivelojosia, Stéphanie Ramboarina, Peter A. Zimmerman, Daniel J. Weiss, Ewan Cameron, Peter W. Gething, Rosalind E. Howes

**Affiliations:** 1 Malaria Atlas Project, Oxford Big Data Institute, Nuffield Department of Medicine, University of Oxford, Oxford, UK; 2 National Malaria Control Programme, Ministry of Health, Antananarivo, Madagascar; 3 University of Antananarivo, Antananarivo, Madagascar; 4 Institut Pasteur de Madagascar, Antananarivo, Madagascar; 5 Faculté des Sciences, Université de Toliara, Toliara, Madagascar; 6 Center for Global Health and Diseases, Case Western Reserve University, Cleveland, OH USA

**Keywords:** Madagascar, *Plasmodium falciparum*, Geostatistical model, Map, Malaria Indicator Surveys

## Abstract

**Background:**

Reliable measures of disease burden over time are necessary to evaluate the impact of interventions and assess sub-national trends in the distribution of infection. Three Malaria Indicator Surveys (MISs) have been conducted in Madagascar since 2011. They provide a valuable resource to assess changes in burden that is complementary to the country’s routine case reporting system.

**Methods:**

A Bayesian geostatistical spatio-temporal model was developed in an integrated nested Laplace approximation framework to map the prevalence of *Plasmodium falciparum* malaria infection among children from 6 to 59 months in age across Madagascar for 2011, 2013 and 2016 based on the MIS datasets. The model was informed by a suite of environmental and socio-demographic covariates known to influence infection prevalence. Spatio-temporal trends were quantified across the country.

**Results:**

Despite a relatively small decrease between 2013 and 2016, the prevalence of malaria infection has increased substantially in all areas of Madagascar since 2011. In 2011, almost half (42.3%) of the country’s population lived in areas of very low malaria risk (<1% parasite prevalence), but by 2016, this had dropped to only 26.7% of the population. Meanwhile, the population in high transmission areas (prevalence >20%) increased from only 2.2% in 2011 to 9.2% in 2016. A comparison of the model-based estimates with the raw MIS results indicates there was an underestimation of the situation in 2016, since the raw figures likely associated with survey timings were delayed until after the peak transmission season.

**Conclusions:**

Malaria remains an important health problem in Madagascar. The monthly and annual prevalence maps developed here provide a way to evaluate the magnitude of change over time, taking into account variability in survey input data. These methods can contribute to monitoring sub-national trends of malaria prevalence in Madagascar as the country aims for geographically progressive elimination.

**Electronic supplementary material:**

The online version of this article (10.1186/s12916-018-1060-4) contains supplementary material, which is available to authorized users.

## Background

Malaria remains an important public health problem in Madagascar despite concerted efforts over decades to control transmission. At times, these interventions, which have been primarily focussed on targeted vector control, have successfully reduced transmission to very low levels [1–3]. The island’s malaria epidemiology is highly varied, reflecting a diverse ecological environment, with differing seasonal trends across the country [4], which can be further affected by unpredictable cyclonic activity that impedes malaria control efforts. Epidemics have punctuated the history of malaria in Madagascar [5]. They are closely associated with the spread of rice production [6] and remain an important component of the disease epidemiology [7]. Domestic political support and international investment following the turn of the century led to impressive reductions in transmission up to 2008, but were followed by a period of stagnation and subsequent reversal of progress in the aftermath of political instability in 2009 and funding disbursement delays [4].

The National Malaria Control Programme (NMCP) of Madagascar has recently entered a new phase, marked by the inauguration of the 2018–2022 National Strategic Plan for Malaria, which includes ambitious targets for progress towards elimination during this period [8]. Central to optimising the intervention policy strategy for the coming 5 years is a thorough understanding of the current epidemiological situation across the country and of the impact of investments in recent years. While routinely reported clinical case data can provide insight into general tendencies in the burden of malaria in Madagascar [4], important uncertainties associated with this stream of data present difficulties with robustly quantifying spatio-temporal trends. Madagascar has benefited from three Malaria Indicator Surveys (MIS 2011, 2013 and 2016), which provide standardised and rigorous measures of malaria endemicity at high spatial resolution from representative population samples from across the island [9–11].

**Fig. 1:**
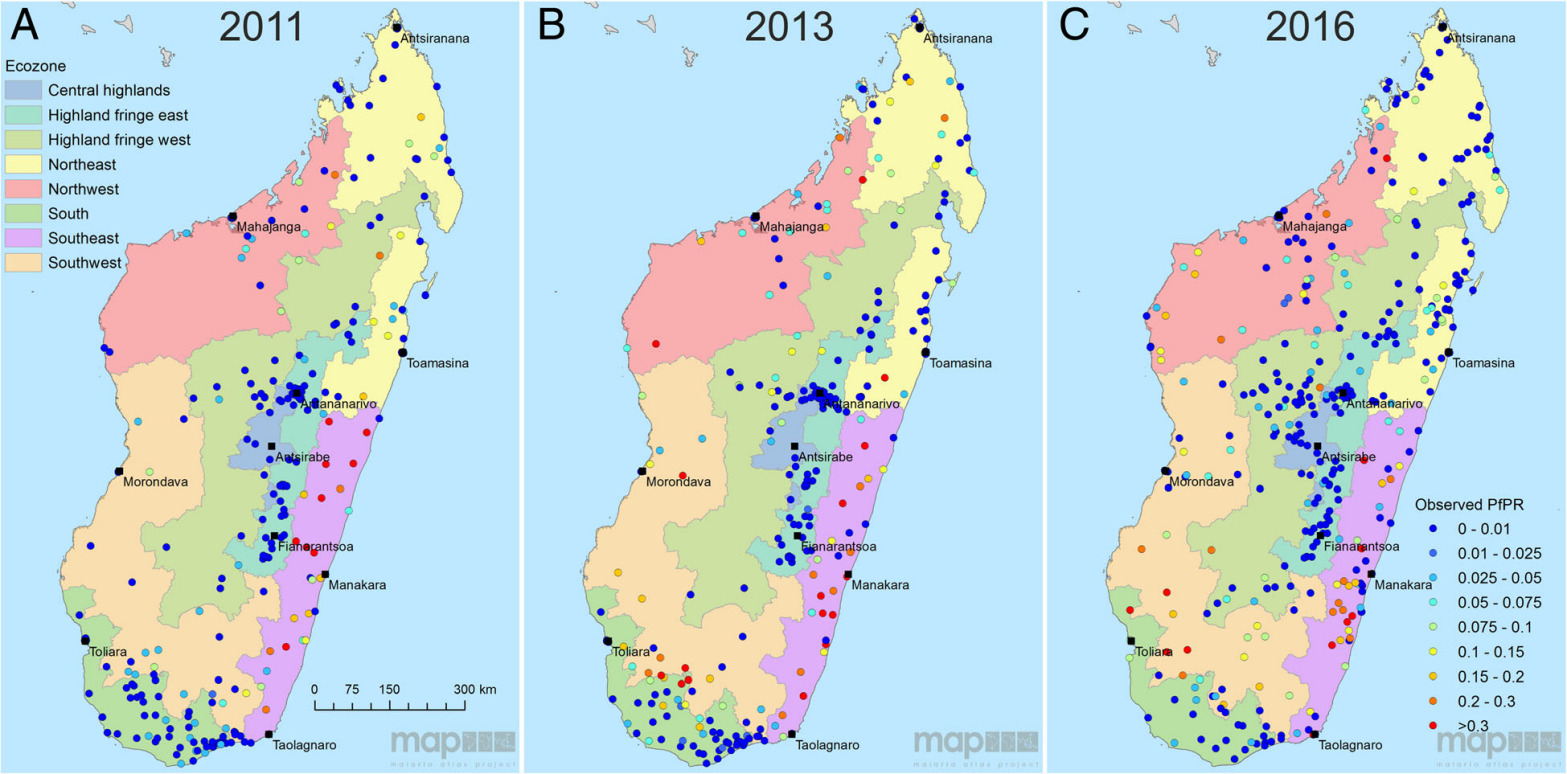
Malaria Indicator Survey screening sites for (**a**) 2011, (**b**) 2013 and (**c**) 2016. The coloured regions represent the country’s eight malaria ecozones [4]

A recent overview of Madagascar’s routine malaria case data from 2010 to 2015 described the trends in reported case data across eight ecozones, which are programmatically relevant clusters of spatially contiguous districts that share similar malaria epidemiological characteristics [4] (Fig. 1). This sub-national perspective offered insight into important variation in malaria burden across the island’s different environmental zones. The overview identified an increase in numbers of confirmed malaria cases. This is explained in part by increased access to rapid diagnostic tests, but also by a growing malaria burden, which is indicated by a significant rate of increase in the diagnostic positivity rate between 2010 (33%) and 2015 (50%) (*p* < 0.0001). The routine reported case data, therefore, indicate there was an increasing malaria burden from 2010 to 2015.

The quality of data reporting through a country’s routine health information system, however, can vary, potentially impacting the reliability of the nationally aggregated health statistics as malariometric indicators, especially in a resource-limited setting such as Madagascar, which has a fragile health and communications infrastructure [12]. At every step of the data reporting chain, cases are lost from the system [13], for example from patients not interacting with public health facilities during malaria episodes [14], rapid diagnostic test stock-outs resulting in unconfirmed infections, or accessibility difficulties for health workers submitting monthly activity reports to the centralised database etc. [4]. Reported numbers can, therefore, be strongly influenced by factors external to the true underlying epidemiology.

MIS datasets provide a complementary perspective on recent trends in malaria endemicity, free from the intrinsic limitations of reported case data. Their strength lies in their standardised methodology applied repeatedly over multiple years across nationally representative sites. This present study uses the MIS data to map the prevalence of infection by *Plasmodium falciparum* in those under 5 years old (6 to 59 months in age: *Pf*PR_6–59mo_) to obtain a deeper insight into epidemiological trends than that available from the published MIS overview analyses, which reported aggregated national prevalence rates of 6.2% for 2011 [9], 9.1% for 2013 [10] and 7.0% for 2016 [11]. This study aims to generate spatially continuous prevalence maps that account for disparities in sample collection dates between the different MIS years. This variation in the time window is not otherwise accounted for in the published MIS analyses [9–11]. The maps provide a benchmark of transmission intensity, free from the nuances of the datasets of routinely reported case data. These can support strategic resource allocation and allow progress towards the current strategic plan goals to be monitored.

## Methods

### Study data

Parasite prevalence data for this study came from MIS activities conducted across Madagascar in 2011, 2013 and 2016. Alongside many other indicators, an MIS includes measurements of the prevalence of *P. falciparum* infection among children 6 to 59 months old (*Pf*PR_6–59mo_), based on a standardised protocol with a stratified sampling strategy across the island that is designed to capture a nationally representative assessment of prevalence. Data are freely available from the Demographic and Health Surveys (DHS) online repository. Across the three MIS years, a total of 898 spatially unique geo-positioned datapoints were available, which reported prevalence of infection among 19,986 children. The initial survey in 2011 included 266 sites (29.6% overall; 6827 individuals), screened from March to May. In 2013, 274 sites (30.5%; 6232 individuals) were screened between April and June. Finally, in 2016, 358 sites (39.9%; 6927 individuals) were screened from April to July. The diagnostic outcomes used here were from microscopy, read by the Institut Pasteur de Madagascar and NMCP parasitology teams. Survey locations are plotted by year in Fig. 1 and by month in Additional file 1: Figure S1. The maps reveal a west to east longitudinal gradient in the cluster sampling in both 2013 and 2016, with the high endemicity east coast sampled later than western and central populations. This sampling bias, with the east coast sampled later in the transmission season, reinforced the need to account for this in the model structure. Full details of the MIS protocols are available in the original published reports [9–11].

Environmental and socio-demographic variables known to interact with and influence *P. falciparum* prevalence [15] were assembled as 30 arcsecond spatial grids (Table 1). Among the suite of covariates, eight were temporally static covariates. The socio-demographic variables included stable night-time lights in 2010 [16], which indicated the presence of cities or towns [17], and accessibility to cities, which quantified travel time to cities larger than 50,000 people [18]. Static environmental variables included elevation as measured from the Shuttle Radar Topography Mission (SRTM) [19], slope, which was calculated from that elevation in ArcGIS 10.5.1 [20], and a measure of distance to water, which indicates the Euclidean distance to permanent and semi-permanent water based on the presence of lakes, wetlands, rivers and streams, and accounts for slope and precipitation [21, 22]. Also included were indices of aridity [23], potential evapotranspiration [23] and topographic wetness (which was calculated from the SRTM elevation surface).

**Table 1:** List of environmental and socio-demographic covariates

Covariate	Description	Dynamic	Round 1 selection	Round 2 selection	Source
Accessibility	Distance to cities with population >50,000	Static	Yes	Yes	Nelson [18]
AI	Aridity index	Static	Yes	Yes	World Clim [23]
DistToWater	GIS-derived surface that measures distance to permanent and semi-permanent water based on presence of lakes, wetlands, rivers and streams, and accounting for slope and precipitation	Static	Yes	Yes	MAP (from WWF surfaces [21, 22])
Elevation	Elevation as measured by the Shuttle Radar Topography Mission (SRTM)	Static	Yes	Yes	SRTM derivative [19]
PET	Potential evapotranspiration	Static	Yes	Yes	Trabucco & Zomer [23]
Slope	GIS-derived surface calculated from SRTM elevation surface	Static	Yes	Yes	MAP (from SRTM [19])
Stable_Lights_2010	Index that measures the presence of lights from towns, cities and other sites with persistent lighting	Static	Yes	Yes	NOAA [16]
TWI	Topographic wetness index	Static	Yes	Yes	MAP (from SRTM [19])
Population size	Estimated population per 1 km × 1 km pixel	Annual	Yes		Linard et al. [24]
EVI	Enhanced vegetation index	Monthly	Lag 0, 3	Lag 3	MODIS derivative [31]
LST_day	Daytime land surface temperature	Monthly			MODIS derivative [30]
LST_delta	Diurnal difference in land surface temperature	Monthly	Lag 0, 1, 2, 3	Lag 0, 1, 2, 3	MODIS derivative [30]
LST_night	Night-time land surface temperature	Monthly			MODIS derivative [30]
TCB	Tasselled cap brightness; measure of land reflectance	Monthly	Lag 0, 2	Lag 2	MODIS derivative [32]
TCW	Tasselled cap wetness	Monthly	Lag 3	Lag 3	MODIS derivative [32]
TSI	Temperature suitability index	Monthly	Lag 0, 1, 2, 3		MAP [26]
CHIRPS	Climate Hazards Group Infrared Precipitation with Station Data	Monthly	Lag 0, 1, 2, 3	Lag 0, 1, 3	CHIRPS [25]

*CHIRPS* Climate Hazards Group Infrared Precipitation with Station Data, *GIS* geographic information system, *MAP* Malaria Atlas Project, *MODIS* Moderate Resolution Imaging Spectroradiometer, *NOAA* National Oceanic and Atmospheric Administration, *SRTM* Shuttle Radar Topography Mission, *WWF* World Wildlife Fund

Temporally dynamic covariates are those for which data were available at monthly intervals, or, for population density, annual intervals [24]. Precipitation data were obtained from the Climate Hazards Group Infrared Precipitation with Station data (CHIRPS) [25]. A *P. falciparum*-specific covariate developed by the Malaria Atlas Project that measures the suitability of air temperature for malaria transmission [26, 27] was included after it was extended to the full study period. The remaining dynamic variables were obtained from Moderate Resolution Imaging Spectroradiometer (MODIS) satellite data [28]. Note that these data were first gap-filled [29] to remove missing values, such as due to cloud cover, before being aggregated to monthly measurements. Three products of temperature data were derived from land surface temperature (LST), including (i) daytime LST, (ii) night-time LST and (iii) the diurnal difference of daytime to night-time LST [30]. MODIS reflectance data were used for measurements of vegetation and moisture: enhanced vegetation index (EVI) [31], tasselled cap brightness (TCB) and tasselled cap wetness (TCW) [32].

The values of the covariate data at the MIS screening locations were extracted for all points. Temporally dynamic covariates were matched to the same month as the survey, as well as 1, 2 and 3 months prior to the survey (lags).

### Bayesian spatio-temporal model

Parasite prevalence data (*Pf*PR_6–59mo_) were modelled via a Bayesian binomial logistic regression model with spatio-temporal random effects accounting for a spatial latent process varying with time. An integrated nested Laplace approximation [33] was adopted for model inference and prediction. The spatio-temporal random effects were modelled using stochastic partial differential equations [34], which represent a Matérn spatio-temporal Gaussian field as a Gaussian Markov random field via triangulation.

Let *Y*_*lt*_, *n*_*lt*_ and *p*_*lt*_ be the number of infected individuals, the number of individuals screened and the prevalence of infection by *P. falciparum* at geocoded location *l* (*l* = 1, …, *N*) for time *t* (*t* = 1, …, *T*). *Y*_*lt*_ is assumed to follow a binomial distribution:

$$ {Y}_{lt}\sim \mathrm{Bin}\left({p}_{lt},{n}_{lt}\right). $$

The prevalence of infection, *p*_*lt*_, is modelled via a linear regression on the logit scale:

$$ \mathrm{logit}\left({p}_{lt}\right)={X}_{lt}^T\boldsymbol{\beta} +f\left(\mathrm{Year}\right)+{\phi}_{lt}. $$

The matrix *X* includes an intercept and a list of environmental and socio-demographic covariates known to affect prevalence. *β* is the vector of regression coefficient, *f*(Year) is a temporal random effect accounting for differences in years in the prevalence data and ϕ_*lt*_ is the spatio-temporally structured random effects. The temporal random effect, *f*(Year), is assigned a first-order autoregressive prior distribution (AR1). Monthly variations in the data are captured via the spatio-temporal process ϕ_*lt*_, which changes in time with an AR1 process modelled at two semi-annual scales, as follows:

$$ {\displaystyle \begin{array}{ll}{\phi}_{l,t}={\varepsilon}_{l1},& \mathrm{if}\;t=1,\\ {}{\phi}_{l,t}=a{\phi}_{l,t-1}+{\varepsilon}_{l,t},& \mathrm{if}\;t=2,\end{array}} $$

where *a* (|*a*| < 1) is a temporal autoregressive coefficient and *ϕ*_*l*, *t*_ is the vector of the spatio-temporally structured effect, which follows a multivariate normal distribution with zero mean and a spatio-temporal covariance function of the Matérn family. See [34] for more details of the specification of the spatio-temporal random field. Since the prevalence data were only available for certain months each year, the AR1 process (time effect) of *ϕ*_*l*, *t*_ is modelled at two half-year intervals to ensure continuity: December to May and June to November.

As part of the variable selection procedure, collinearity among the environmental covariates was examined by calculating variance inflation factors (VIFs) prior to fitting the statistical model to the prevalence data. A stepwise selection of covariates using VIFs was undertaken to make sure all VIF values are below a desired threshold (VIF < 10 in this case). Put simply, using the full set of covariates, a VIF for each variable was calculated. The variable with the single highest value was removed and all VIF values with the new set of variables were recalculated. Then, the variable with the next highest value was removed, and so on, until all VIF values were below the threshold of 10. This resulted in a set of 26 covariates being considered for model fitting (Table 1, Round 1 selection). Subsequently, variable selection was performed using bidirectional elimination by running stepwise regressions on all model combinations using the 26 chosen covariates. The best-fitting (final) model had the smallest deviance information criterion value and included 18 covariates (Table 1, Round 2 selection).

Using the final model, the prevalence of infection by *P. falciparum* (*Pf*PR_6–59mo_) was predicted over a 30 arcsecond spatial grid of 1,602,342 pixels (approximately 1 km × 1 km spatial resolution) for each month of 2011, 2013 and 2016. The final covariates were available at monthly intervals and this allowed us to make monthly predictions of prevalence. The resulting predictions included the monthly mean prevalence and the monthly interquartile range (IQR) of prevalence as a measure of the associated prediction uncertainty. The annual mean prevalence (*Pf*PR_6–59mo_) and mean IQR were obtained by averaging across the 12 monthly predictions.

This Bayesian model-based geostatistical approach allows the smoothing of extreme rates due to small sample sizes by borrowing strength across neighbouring pixels to improve local estimates. Essentially, the approach assumes a positive spatial correlation between observations, borrows more information from neighbouring pixels than from pixels far away (in both space and time), and smooths local rates toward local, neighbouring values. The resulting smoothed prevalence estimates are robust and reliable while circumventing the issue of sparse data [35]. The inherently hierarchical structure of the current modelling framework permits the model-based estimation of covariate effects, the prediction of missing data and the estimation of spatio-temporal covariance structures [36]. Model predictions are presented at the pixel level and also summarised by ecozone [4].

A range of model validation analyses were used to assess the model’s goodness of fit and predictive accuracy, including the Pearson correlation of observed and predicted data, and validation runs with incremental hold-out validation data subsets.

## Results

### The model framework

The Bayesian spatio-temporal model was developed in an integrated nested Laplace approximation framework to map the prevalence of *P. falciparum* malaria infection among children aged 6 to 59 months in age (*Pf*PR_6–59mo_) across Madagascar for 2011, 2013 and 2016. Validation of the final model gave Pearson correlation coefficients of 0.96, 0.94 and 0.83 for each prediction year, respectively, between month-specific predicted and observed prevalence (Additional file 2: Figure S2), justifying a good fit of the Bayesian spatio-temporal model to the observed prevalence data. To assess the predictive power of the specified model, prevalence data were split into two sets: a training set (*D*_*t*_) and a validation set (*D*_*v*_) [37]. After being trained on *D*_*t*_, the model accuracy was determined by its ability to predict *D*_*v*_ as well as the entire prevalence dataset. A percentage of *D*_*v*_ was chosen to be a random sample of 10%, 20%, 30% and 40% of the overall dataset. Each validation was repeated 100 times and the model accuracy was averaged across the 100 fitted models. The box plots of cross-validated correlations appeared satisfactory (Additional file 3: Figure S3a). The cross-validated *R*^2^ had a mean of 0.78 and a range of (0.70, 0.83) for 10% *D*_*v*_; a mean of 0.72 and a range of (0.62, 0.79) for 20% *D*_*v*_; a mean of 0.65 and a range of (0.55, 0.74) for 30% *D*_*v*_; and a mean of 0.58 and a range of (0.45, 0.68) for 40% *D*_*v*_ (Additional file 3: Figure S3b). The model was deemed accurate in its ability to predict a wide range of validation sets (Additional file 4: Figure S4).

### Covariates

The combination of covariates that together best explained the spatio-temporal variation in the *Pf*PR datapoints were selected for inclusion in the spatio-temporal model and are listed in Tables 1 (Round 2 selection) and 2. Out of the 18 predictors which informed the statistical model, eight predictors were significant based on their 95% Bayesian credible intervals, namely LST_delta_2, TWI, PET, CHIRPS_1, Accessibility, Elevation, CHIRPS_0 and TCW_3. Of these eight predictors, LST_delta_2 (diurnal variation in land surface temperature 2 months prior to the prediction month) had a positive effect on the odds of risk with odds ratio OR = 1.1987. The odds of *P. falciparum* infection were negatively associated with TCW_3 (an index of surface wetness 3 months prior to the prediction month) with OR = 0.0025. The remaining six covariates had OR slightly above or below 1, indicating that they did not have a large influence on the odds of risk. Note that the final model also included several predictors with OR being roughly one, namely aridity index, distance to water and slope, which suggests that they did not affect the odds of *P. falciparum* infection. However, the model selection procedure that optimised the deviance information criterion value justified the need to retain these predictors in the model.

**Table 2:** Regression coefficients and odds ratios of the predictors selected by the final model and their associated 95% Bayesian credible intervals (CI)

Covariate	Regression coefficient	95% CI of regression coefficient	Odds ratio	95% CI of odds ratio
EVI_3	1.5872	(− 0.3861, 3.5926)	4.8913	(0.6799, 36.3362)
LST_delta_2	0.1812	(0.1098, 0.2527)*	1.1987	(1.1161, 1.2876)*
TWI	0.0535	(0.0048, 0.1017)*	1.0550	(1.0048, 1.1071)*
PET	0.0028	(0.0009, 0.0048)*	1.0028	(1.0009, 1.0048)*
CHIRPS_1	0.0023	(0.0010, 0.0037)*	1.0023	(1.0010, 1.0037)*
Accessibility	0.0020	(0.0009, 0.0031)*	1.0020	(1.0009, 1.0031)*
CHIRPS_3	0.0004	(−0.0010, 0.0018)	1.0004	(0.9990, 1.0018)
AI	0.0001	(0.0000, 0.0002)	1.0001	(1.0000, 1.0002)
DistToWater	0.0000	(0.0000, 0.0001)	1.0000	(1.0000, 1.0001)
Slope	0.0000	(0.0000, 0.0000)	1.0000	(1.0000, 1.0000)
Elevation	−0.0015	(−0.0022, − 0.0009)*	0.9985	(0.9978, 0.9991)*
CHIRPS_0	−0.0020	(−0.0036, − 0.0004)*	0.9980	(0.9964, 0.9996)*
LST_delta_1	−0.0111	(−0.0944, 0.0721)	0.9889	(0.9099, 1.0748)
Stable_Lights_2010	−0.0274	(−0.0801, 0.0218)	0.9730	(0.9231, 1.0221)
LST_delta_3	−0.0532	(−0.1173, 0.0106)	0.9482	(0.8894, 1.0106)
LST_delta_0	−0.0540	(−0.1360, 0.0281)	0.9475	(0.8729, 1.0285)
TCB_2	−1.7339	(−5.2983, 1.8037)	0.1765	(0.0050, 6.0672)
TCW_3	−5.9884	(−10.7712, − 1.2680)*	0.0025	(0.0000, 0.2813)*
Intercept	−11.5150	(−15.6992, −7.5190)	0.0000	(0.0000, 0.0005)

*CI* credible interval

*Significant based on 95% Bayesian credible interval

**Fig. 2:**
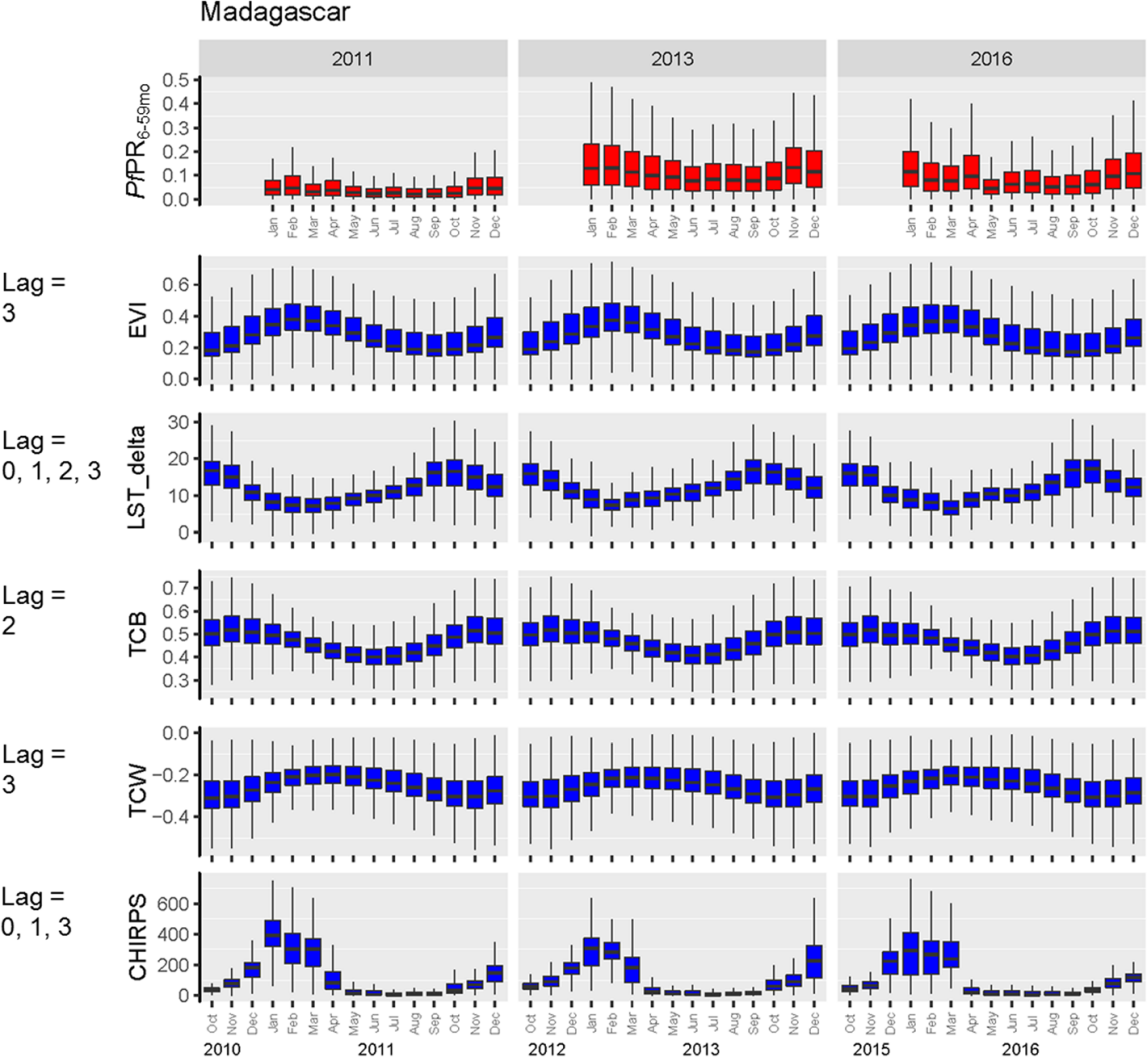
National-level mean monthly *Pf*PR_6–59mo_ predictions, plotted alongside temporally variable predictor values. The box plot rectangles indicate the first to third quartiles (interquartile range), with the median shown as the dark line inside the box. Vertical lines correspond to the minimum and maximum values. Specified lags indicate the time points that were selected by the model as explanatory variables of *Pf*PR_6–59mo_. A time lag of 0 indicates that the covariate values in the concurrent month were predictive of *Pf*PR_6–59mo_, while a time lag of 3 indicates that the covariate value 3 months prior to the *Pf*PR_6–59mo_ prediction was predictive of *Pf*PR_6–59mo_

Monthly variation in the key predictors is summarised at the national level in Fig. 2 and by the previously defined ecozones [4] in Additional file 5: Figure S5. These box plots include the temporally dynamic environmental predictors that informed the statistical model over different time lags. For example, EVI with a 3-month lag indicates that the malaria prevalence prediction for January 2011 was informed by the EVI level in October 2010. LST_delta, or the diurnal variation in land surface temperature, was influential across the whole time window up to 3 months prior to the prediction months. Predictions for January 2011, for example, were influenced by the difference in daytime and night-time temperatures (LST_delta) in October, November and December 2010, as well as January 2011. Seasonal variation in the different dynamic predictors differed in amplitude, with the most extreme being the CHIRPS dataset (a precipitation indicator), which dropped to very low between May and September. In contrast, the tasselled cap wetness (a remotely sensed transformed index of land surface water availability) remained relatively constant over time, with no significant monthly variation when summarised to the national level. At the higher-resolution ecozone level, ecological predictors show more extreme temporal variation, emphasising the country’s environmental diversity. Put together, these constellations of monthly dynamic explanatory variables combined with the static predictors explained the spatio-temporal variation in the input data and informed extrapolation to locations and time points that were not represented in the MIS input datasets.

### Spatio-temporal trends in malaria endemicity

**Fig. 3:**
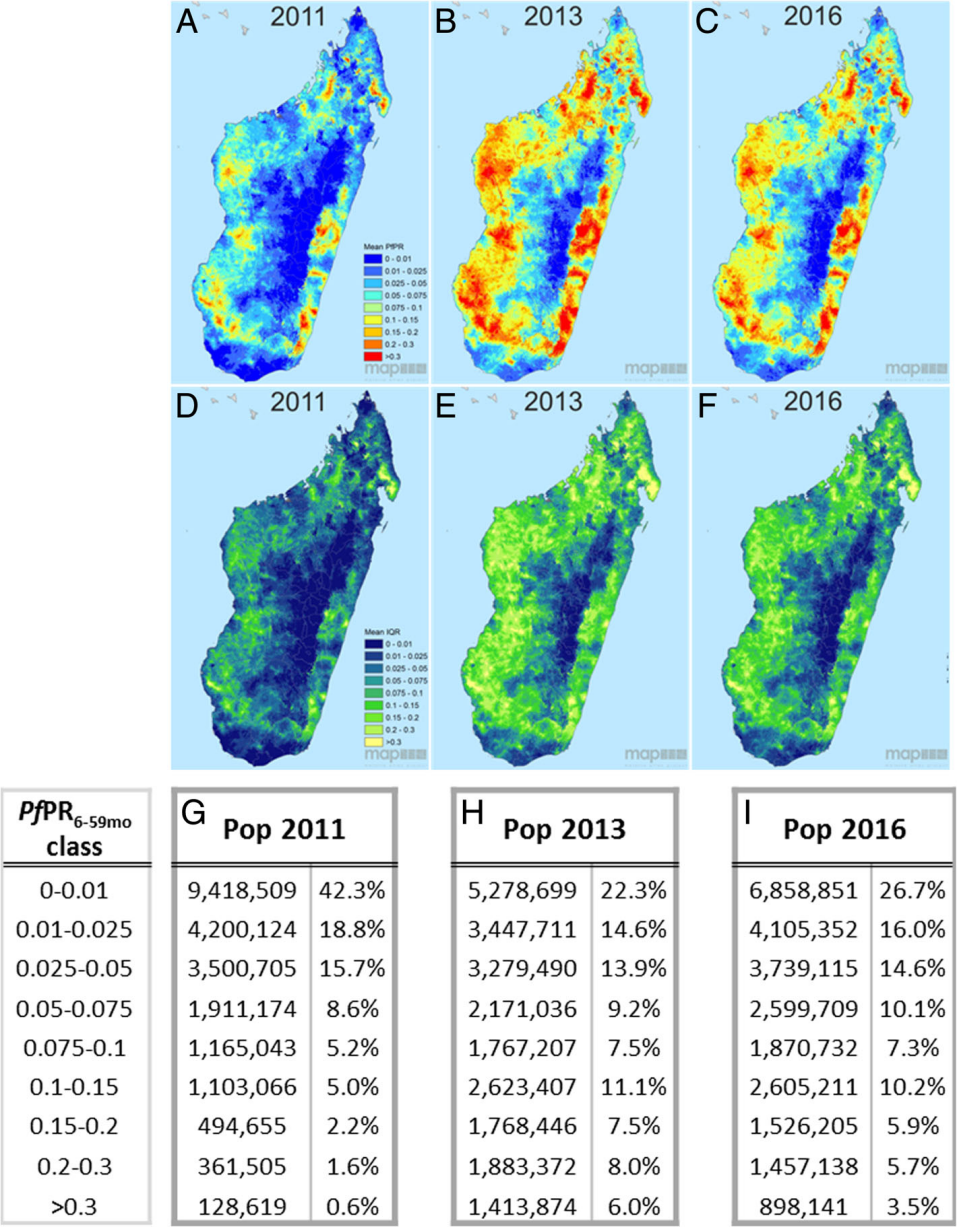
Predicted annual mean *Pf*PR among children 6 to 59 months in age for 2011 (**a**), 2013 (**b**) and 2016 (**c**). **d**–**f** The corresponding map uncertainty (quantified as the prediction interquartile range). Values are mapped at 1 × 1 km pixel resolution. **g**–**i** National population breakdown by endemicity class, using population values based on WorldPop’s Whole Continent UN-adjusted Population Count datasets for Africa for 2010, 2015 and 2020. Estimates for 2011, 2013 and 2016 were created by linear interpolation of the bookending quinquennial rasters

**Fig. 4:**
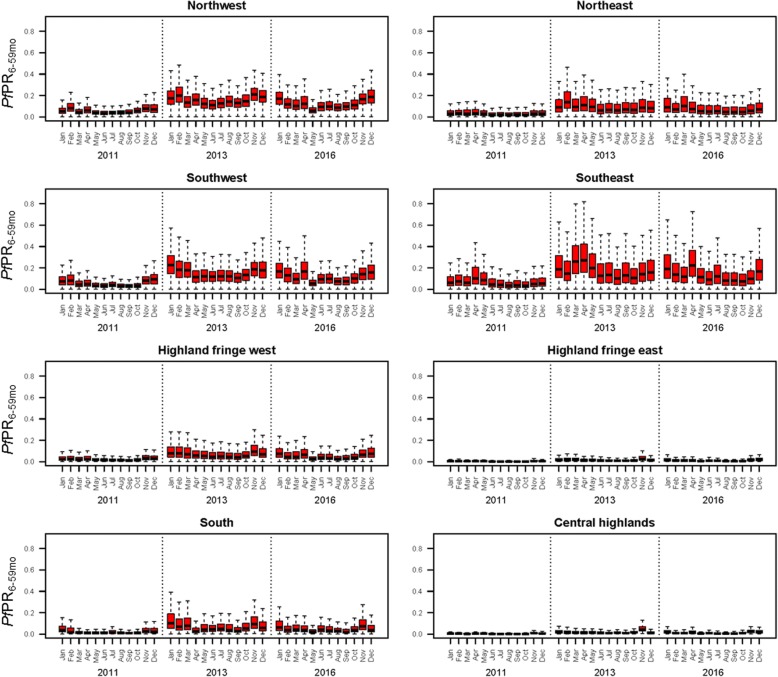
Box plots of predicted monthly *Pf*PR_6–59mo_ by ecozone for 2011, 2013 and 2016. Ecozone extents are shown in Fig. 1 [4]

This paper’s primary objective was to assess changes in the prevalence of *P. falciparum* malaria infection among those under 5 years old (*Pf*PR_6–59mo_) in Madagascar between the three surveyed years of 2011, 2013 and 2016. The published MIS reports gave national prevalence estimates for the 6–59 month age group of 6.2% [9], 9.1% [10] and 7.0% [11], respectively. The geostatistical model developed here generated 36 monthly prediction maps of endemicity (Additional file 5: Figure S5 and Additional file 6: Figure S6), which are summarised into three annual mean predictions (Fig. 3a–c), with ecozone-level box plot summaries of trends across the country’s eight ecozones given in Fig. 4. Together, these model outputs allow spatio-temporal trends to be examined at different spatial and temporal scales: national, ecozone or pixel level, and monthly or annually. Model uncertainty is mapped alongside the mean *Pf*PR_6–59mo_ prediction surfaces as the IQR of the model posterior distribution (Fig. 3d–f and Additional file 6: Figure S6b, d, f), quantifying confidence in the model predictions. Coefficients of variation (standard deviation/mean) for each annual prediction are plotted in Additional file 7: Figure S7 to reflect the relative variability of the predictions. Together, these uncertainty maps show that while the absolute uncertainty (represented by the IQR) is lower in the central highlands where prevalence is lowest, when adjusted to the mean prediction values, confidence in the prediction is strongest in coastal areas where prevalence is higher.

Malaria endemicity remains spatially heterogeneous across Madagascar (Figs. 3 and 4), with the central highlands consistently having the lowest endemicity across the 3 years evaluated (<1% mean annual *Pf*PR_6–59mo_). To the east, a sharp transition towards the coast culminates in high prevalence regions, with pockets along the south-east coast with >30% mean annual prevalence, expanding in extent from 2011 into subsequent years. High prevalence in this ecozone is not homogeneous however, being interspersed with pockets of lower endemicity (<7.5% *Pf*PR_6–59mo_). The island’s west coast is similarly high in prevalence, almost universally with *Pf*PR_6–59mo_ > 10% and with areas >30% mean annual *Pf*PR_6–59mo_ prevalence. The northern and southern tips have lower prevalence, typically under 5% endemicity. Confidence in the model predictions was variable across the country and between years. The low-prevalence data-rich highlands were modelled with greater confidence than the more heterogeneous coastal regions, where survey data indicated spatially variable rates of infection (Figs. 1 and 3d–f). The highest model uncertainty in all 3 years was in the north-eastern region of Sava, an area previously characterised as a malaria parasite transmission hotspot [38]. As seen across the whole country after 2011, the higher observed prevalence was associated with a larger IQR, reflecting the wider range of potential prevalence values.

**Fig. 5:**
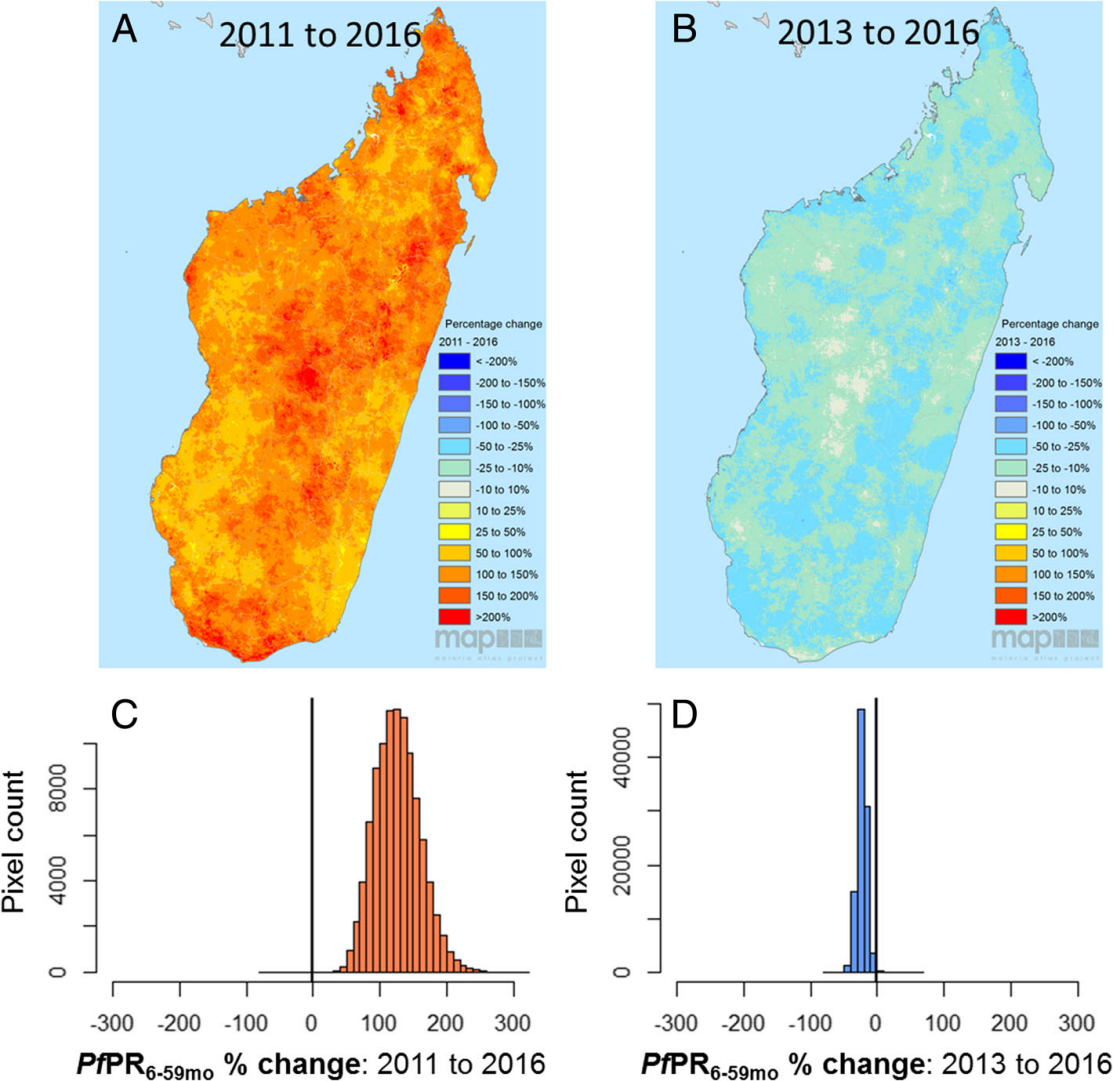
Percentage changes in predicted *Pf*PR among children 6 to 59 months old across the three MIS time points: **a** from 2011 to 2016 and **b** from 2013 to 2016. Histograms of pixel-level change **c** from 2011 to 2016 and **d** from 2013 to 2016. Positive % change indicates an increase in prevalence, while negative % change is a decrease

At the national level, the three mean annual maps were all significantly different from one another (Wilcoxon rank sum test, *p* < 2.2 × 10^-16^). *Pf*PR_6–59mo_ more than doubled between 2011 and 2016 (127% increase across the mean annual maps; Fig. 5a and c), despite a 23% decrease within that window between 2013 and 2016 (Fig. 5b and d). Changes in endemicity were spatially variable across the period examined. The magnitude of change across the country between 2011 and 2016 was highly heterogeneous, with a standard deviation of 34.0% around the 127% mean increase. All ecozones experienced at least a doubling in prevalence of *Pf*PR_6–59mo_ between 2011 and 2016, with the smallest proportional change being in the south-east (100.4%; Fig. 4 and Additional file 5: Figure S5d) and the highest up to 157.4% in the central highlands, where prevalence nevertheless remained the lowest (Fig. 4 and Additional file 5: Figure S5h). The south ecozone also saw an important increase of 142.5% over the 6-year period (Fig. 4 and Additional file 5: Figure S5g). The 23% mean fall in prevalence from 2013 to 2016 was fairly consistent across the whole country (standard deviation 7.4%), and at the ecozone level, ranged from a 20.7% decrease in the central highlands to a 27.4% decrease in the eastern highland fringe zone. At the pixel level, 96% of pixels experienced a decrease of 10% to 50% between 2013 and 2016. In contrast, 27% of pixels increased between 0% and 100%, and 71% more than doubled in prevalence over the full study period from 2011 to 2016.

### Trends in population exposure

Converting the geographic maps into population exposure rates offers insight into the relative intensity of infection prevalence across the population. The density of which is highly variable across the island [4]. Fig. 3g–i summarises the population numbers resident in each of the different endemicity categories. These show that the majority of the Malagasy population lives in the lowest endemicity areas, with almost half (42%) of the population in very low endemicity areas (<1% mean annual *Pf*PR_6–59mo_) and less than 10% in areas where *Pf*PR_6–59mo_ was >10% in 2011. The highest endemicity year, 2013, was predicted to have 32.5% of the population in areas where endemicity was >10%, a threefold increase from 2011. By 2016, this had dropped to 25.3% of the population being in areas of >10% prevalence, with a corresponding increase in the proportion living in very low (<1%) prevalence areas to 26.7%. Finally, the proportion of the population with infection prevalence greater than 20% rose from 2.2% in 2011 (an estimated 0.5 million individuals) to 14.0% in 2013 (3.3 million individuals) and 9.2% (2.4 million individuals) in 2016. Trends in endemicity across the population, therefore, reflect the overall tendencies in the geographic maps, with sharp increases in infection prevalence in the low endemicity areas where most of the population lives, and notable increases in the population exposed to the highest end of the endemicity spectrum. A decrease of exposure levels from 2013 to 2016 was evident, but much less pronounced than the increase between 2011 and 2013, meaning that the exposure levels in 2016 were considerably worse than in 2011.

## Discussion

Three MIS studies have taken place in Madagascar since 2011, providing a valuable source of information about the status of malaria from a standardised data collection protocol applied across a nationally representative set of locations. A broad range of indicator metrics are included in the MIS reports. This present study looks at the parasitology data in particular to assess how the prevalence of malaria infection in children 6 to 59 months old has changed in recent years. Given the limitations of the routine health data reporting chain discussed previously [4], this study provides a complementary perspective into the recent status of malaria endemicity in Madagascar, identifying important increasing trends since 2011 despite a relatively small fall in prevalence between 2013 and 2016.

### Comparison with reported MIS results

The results presented in MIS reports are summaries of the raw survey data, with individuals weighted in such a way as to ensure appropriate national representation in the overall summary statistics. However, the results are not adjusted for the temporal lag in survey dates across years (Additional file 1: Figure S1). By 2016, the survey time window was pushed to 2 months later than in 2011, corresponding to a delay away from the peak transmission period in most regions (Additional file 3: Figure S3 and Additional file 4: Figure S4). The model presented here specifically accounts for this temporal lag, allowing for meaningful comparisons between years. Seasonal trends in transmission [4] mean that a delayed survey period will underestimate endemicity relative to the original survey time period. This is reflected in the raw MIS results, which suggest a 6.2% national *Pf*PR_6–59mo_ in 2011 and 7% in 2016, a 13% increase over that period. In contrast, the spatio-temporal model presented here identifies a 127% increase, resulting in an estimated national mean 9.3% annual *Pf*PR_6–59mo_ in 2016.

The MIS specifically excludes three highland districts considered to be malaria-free (the cities of Antananarivo-Renivohitra, Antsirabe I and Fianarantsoa I) as well as communes above 1500 m in altitude. Despite not being represented in the mapping input dataset, model predictions are derived for these areas, which then inform the ecozone- and national-level summaries. Without prior exclusion of these zones, the model-based approach risked over-inflating the estimates of endemicity. Environmental covariates, however, appear to have discerned the unsuitability of the highland urban habitat, and the malaria-free districts are predicted to have <0.5% infection prevalence in all 3 years. The overall ecozone-level mean annual *Pf*PR_6–59mo_ for the central highlands was 0.6% in 2011, 2.0% in 2013 and 1.6% in 2016, which were of the same order of magnitude as those in the MIS summaries of 0.8%, 1.1% and 0.9%, respectively. The excluded MIS sampling zones do not, therefore, seem to have impacted the spatio-temporal model predictions.

### Comparison with previous prevalence maps

While geostatistical methods have been previously applied to infection prevalence datasets to predict spatially continuous prevalence maps of Madagascar, this has been within the context of continental or global-level mapping [37, 39]. The country-specific modelling approach applied here allows more freedom to the environmental covariates to adapt to Madagascar’s specific ecological context, with the model selecting those covariates most pertinent to the island’s environmental diversity. The continent-level spatio-temporal prediction cube developed by Bhatt et al. ([39], reproduced for Madagascar [4]), indicates a much coarser granularity than the present predictions, with little variation between years. Nevertheless, the continental maps do allow malaria endemicity in Madagascar to be viewed in its broader context, showing the relatively low prevalence of infection in Madagascar relative to many countries in sub-Saharan Africa (incidence rate ranked 13th lowest out of 43 countries in 2015 [39]). Prevalence mapping analyses are also valuable in evaluating trends in malaria prevalence over time.

### Comparison with health metrics information system data

In this study, we map the parasite reservoir, as detectable by microscopy. In parallel, clinical case numbers are collated by the routine health metrics information system [4, 40, 41]. While the two metrics report different characteristics of malaria, spatio-temporal trends from both are similar, with a comparable geographic distribution of the burden of disease and an important increase in burden particularly along the west coast. A major increase in clinical cases was reported between 2014 and 2015, which subsequently reduced in 2016 (the third MIS year) following a mass distribution of bed nets treated with insecticide at the end of 2015 [41]. The longitudinal nature of the data from the health metrics information system allows extreme events to be identified, such as epidemics, which may drastically affect the annual case totals, as in 2015, but which may not be distinguishable as exceptional, or even captured by cross-sectional surveys that assess prevalence at isolated time points.

A 2017 World Health Organization (WHO) report estimates that only around 31% of all clinical cases are reported through the surveillance system to the central level in Madagascar. This is likely primarily driven by the population’s low rates of seeking treatment. Of mothers seeking treatment in 2016 for their febrile children aged 6 to 59 months, 35.8% did so at public health facilities and 46.2% did so at any source (including public health facilities) [11]. Such low capture of the overall case burden, therefore, throws into question the system’s capacity to adequately quantify changes in the malaria burden over time.

The two indicators, therefore, have their strengths and limitations, but together corroborate general trends of an increased burden between 2011 and 2016, punctuated with reductions, such as those identified between the 2013 and 2016 MIS surveys, or between 2015 and 2016 by the routinely reported case data [40, 41]. This temporal heterogeneity may be partly attributable to reductions in NMCP activities caused by Global Fund disbursement delays in 2014 [40]. In addition, recent evidence of the variable quality and durability of the insecticide-treated bed-net brands distributed across the country means that over their 3-year lifespans (mass distribution campaigns in Madagascar are triennial), protection from nets will be inconsistent [42]. MIS campaigns in Madagascar have all been timed to take place in the transmission season following mass bed-net distribution campaigns, which may, therefore, capture snapshots of prevalence at its lowest.

### Limitations to the approach

In this study, we have used data on the prevalence of infection to assess the current spatio-temporal trends of malaria infection in Madagascar. This metric is independent of clinical symptoms, and instead quantifies the extent of the parasite reservoir across the population. The value of such a metric lies in its simplicity and standardised collection methods, and, in the context of the MIS, repeated national representation. A recent review by Cohen and colleagues [43], however, has argued for a multi-component mapping process that will adequately identify the underlying drivers of transmission, to enable NMCP to target control measures optimally. Elimination requires both the reduction of the parasite reservoir and the prevention of transmission. Prevalence data alone cannot fully characterise the local epidemiology without a parallel understanding of a site’s historical context, the significance of imported cases, the impact of recent control efforts, entomological and host behavioural/genetic factors, and so on [43]. A practical interpretation of the prevalence map, therefore, requires insight into the underlying factors driving the observed infection rates. The map suite presented here is one component in understanding malaria in Madagascar, but ought to be interpreted in association with a broader set of malariometric data when used to determine control intervention policy. Malaria transmission is also highly dynamic both spatially and temporally, meaning that predicted maps based on single time-point snapshots of prevalence may simplify the true underlying situation. Maps such as those presented here provide insight into general trends, and the validation statistics presented in Additional file 2: Figure S2, Additional file 3: Figure S3 and Additional file 4: Figure S4 indicate the level of variability that might be expected around these predictions.

Malaria prevalence is strongly influenced by intervention coverage levels, including rates of insecticide-treated net (ITN) ownership [39] and treatment seeking [13, 14]. Including these covariates in the modelling framework could help inform the model about the patterns of endemicity but this was not considered feasible in this present analysis. The coverage of indoor residual spraying and ITN use rates, for instance, have complex non-linear relationships with malaria prevalence. For example, indoor residual spraying in Madagascar is carefully targeted to the highest (as an emergency response to reduce mortality during outbreaks) and lowest (prevention of reintroduction and subsequent autochthonous transmission) endemicity districts only [8]. ITN coverage is strongly skewed to areas where malaria is endemic. The highland areas into which malaria is mainly imported are not covered by routine ITN distribution. The limited temporal window considered in this present study does not allow for the protective effect of high ITN coverage to be learnt by the model, and instead the coarse learnt association is that ITN coverage increases as prevalence increases. Treatment seeking was excluded for reasons of sample sizes. MIS data on treatment seeking from individual cluster locations are limited to mothers with infants who suffered from fever in the 2 weeks preceding the MIS interviews. Sample sizes at the cluster level are, therefore, very small, producing spurious results when analysed at the high resolution of the present analysis. Despite these barriers to including intervention covariates in the model, the suite of environmental and socio-demographic variables that were used allowed robust predictions of malaria prevalence, so this was not considered a major limitation to the mapping model presented here.

Madagascar is noted for its mosaic landscape of ecological habitats, with land cover varying across short distances [44]. The critical importance of the environmental covariates in the modelling process is evident from the methods described here, with a strong predictive role associated with vegetation cover that explains differences in malaria prevalence between different areas (Table 2). The covariates associated with each MIS site describe the local conditions associated with the observed malaria prevalence at the time and location of sampling. However, MIS datasets are geopositioned with a deliberate degree of spatial uncertainty (displacement) to promote anonymity of up to 2 km in urban areas, 5 km in rural areas and 10 km for 1% of rural points [45]. This spatial displacement, therefore, introduces uncertainty into the associations between reported *Pf*PR and their attributed covariate values, which could impact the model’s predictions. For this study, we assumed that while the island is ecologically heterogeneous, the impact of this spatial uncertainty will be acceptably low (with ecological conditions similar for most points even at a distance of 5 km or 10 km), and similar enough to allow a prevalence signal to be identified. The model validation statistics corroborate this assumption.

A further limitation of the MIS datasets that informed the current mapping analysis stems from their sampling design and sample sizes. The broad range of indicators included in the MIS activities present conflicting demands on sample sizes, which are further constrained by financial and logistical considerations. Sample sizes cannot, therefore, be optimised for all indicators, but instead are focussed on a limited number of these. A recent retrospective model-based analysis of the 2011 and 2013 Madagascar MIS datasets estimated that sample sizes were under-powered by 17% and 36%, respectively, to reach effective sizes for infection prevalence rates [46]. This was based on rapid diagnostic test results and not microscopy (as considered in this present study), meaning that it may be a slight overestimate. The approach followed here, namely to consider samples from the three MIS datasets within a common Bayesian hierarchical modelling framework and to draw on a broad range of associated covariate surfaces, provides a solution to sample size limitations.

Furthermore, at very low transmission levels, such as in the central highlands and parts of the central fringe regions of Madagascar, microscopy-based cross-sectional surveys of parasite prevalence risk are underpowered to detect rare and low parasitaemia infections adequately [47]. In these areas, higher sensitivity diagnostics (such as nucleic-acid amplification-based approaches) and alternative indicators (such as serological markers) are required to monitor malaria epidemiological trends more effectively [47–50]. Particular interest is in the use of serological panels targeting both short- and long-lasting antigenic responses [51]. When applied to appropriate sentinel populations [52, 53], these tools can be powerful probes of changing transmission intensity or reintroduction events in the pre-elimination setting where the majority of infections at the time of survey may be microscopically sub-patent [54].

## Conclusions

Malaria remains an important health problem in Madagascar, with the prevalence of infection more than doubling between 2011 and 2016 (a mean increase of 127%). Across the three survey time points, 2011 to 2013 saw the greatest *Pf*PR_6–59mo_ increase, followed by a reduction to 2016 (mean reduction of 23%). However, while the whole population is at risk of infection, prevalence was lower in higher density areas, with 26.7% of the population in 2016 living in pre-elimination areas, where prevalence was <1% (a notable reduction from 42.3% of the population in 2011, however).

Presidential elections in December 2013 marked an optimistic turning point for Madagascar, with the return to democracy ending the country’s 5-year isolation from the international community. The 2009–2013 political crisis placed a heavy burden on the country’s socio-economic situation, with a deterioration in infrastructure and public services [55]. The sharp increase in malaria prevalence observed from 2011 to 2013 is likely a consequence of the country’s wider economic situation and associated health infrastructure breakdown.

Madagascar is not alone in suffering losses with malaria control, with most countries in the African WHO region also experiencing stalling progress [40]. Madagascar’s new strategic plan for 2018–2022, however, offers an opportunity to strengthen control with a policy shift away from blanket coverage of intervention commodities, towards a more locally targeted programme responsive to specific epidemiological contexts [56]. The current government’s strong support for malaria elimination is reflected by a move towards closer integration of the NMCP into the Ministry of Health’s core structures and activities rather than being a quasi-independent programme, coupled with the objective of partially devolving responsibilities for control planning to regional officers. New policies include the reintroduction of entomological control interventions, targeted seasonal chemoprophylaxis in epidemic-prone south-western communities, expanding household insecticide residual spraying to the highest and lowest risk areas, and specific consideration of high-risk populations [8].

Ambitious targets are being set for the end of the next National Strategic Plan in 2022, with a view towards geographically progressive elimination. Prevalence maps, as presented here, represent one component of monitoring progress towards those goals.

## Additional files

Additional file 1: **Figure S1.** Sample screening during the three MIS events in Madagascar, showing the progressive delay in the sampling time window. a Overall bar plots of sampling months. b–d Maps by sampling month by cluster location. (PNG 1353 kb)

Additional file 2: **Figure S2.** Month-specific correlations between observed raw MIS prevalence values and the model predictions for each annual map. Pearson correlation coefficients are shown on each plot. (PNG 32 kb)

Additional file 3: **Figure S3.** Box plots of a cross-validated Pearson correlation coefficients and b cross-validated *R*^2^, based on 100 randomly sampled validation sets. (PNG 26 kb)

Additional file 4: **Figure S4.** Observed prevalence against predicted prevalence averaged across 100 randomly sampled validation sets. (PNG 63 kb)

Additional file 5: **Figure S5.** Summary box plots of predicted monthly *Pf*PR_6–59mo_ by ecozone, plotted alongside temporally variable predictor values. The box plot rectangles indicate the first to third quartiles (interquartile range), with the median shown as the dark line inside the box. Vertical lines correspond to the minimum and maximum values. Specified lags indicate the time points that were selected by the model as explanatory variables of *Pf*PR_6–59mo_. A time lag of 0 indicates that the covariate values in the concurrent month were predictive of *Pf*PR_6–59mo_, while a time lag of 3 indicates that the covariate value 3 months prior to the prediction was predictive of *Pf*PR_6–59mo_. (ZIP 1859 kb)

Additional file 6: **Figure S6.** Predicted monthly mean *Pf*PR_6–59mo_ maps for a 2011, c 2013 and e 2016, with associated uncertainty (interquartile range) for b 2011, d 2013 and f 2016. (ZIP 48310 kb)

Additional file 7: **Figure S7.** Maps of the coefficient of variation in the annual mean maps (standard deviation/annual mean) showing the relative confidence in the predictions across the country for a 2011, b 2013 and c 2016. (PNG 535 kb)

## Abbreviations

AR1: First-order autoregressive prior distribution
CHIRPS: Climate Hazards Group Infrared Precipitation with Station Data
CI: Credible interval
DHS: Demographic and Health Surveys
EVI: Enhanced vegetation index
GIS: Geographic information system
IQR: Interquartile range
ITN: Insecticide-treated net
LST: Land surface temperature
MAP: Malaria Atlas Project
MIS: Malaria Indicator Survey
MODIS: Moderate Resolution Imaging Spectroradiometer
NMCP: National Malaria Control Programme
NOAA: National Oceanic and Atmospheric Administration
OR: Odds ratio
*P.*: *Plasmodium*
SRTM: Shuttle Radar Topography Mission
TCB: Tasselled cap brightness
TCW: Tasselled cap wetness
VIF: Variance inflation factor
WHO: World Health Organization
WWF: World Wildlife Fund
