# Supplementary figures and images for: Spatio-temporal mapping of Madagascar’s Malaria Indicator Survey results to assess Plasmodium falciparum endemicity trends between 2011 and 2016
Source: BMC Med. 2018 May 23;16:71. doi: 10.1186/s12916-018-1060-4 (PMC5964908; doi:10.1186/s12916-018-1060-4)

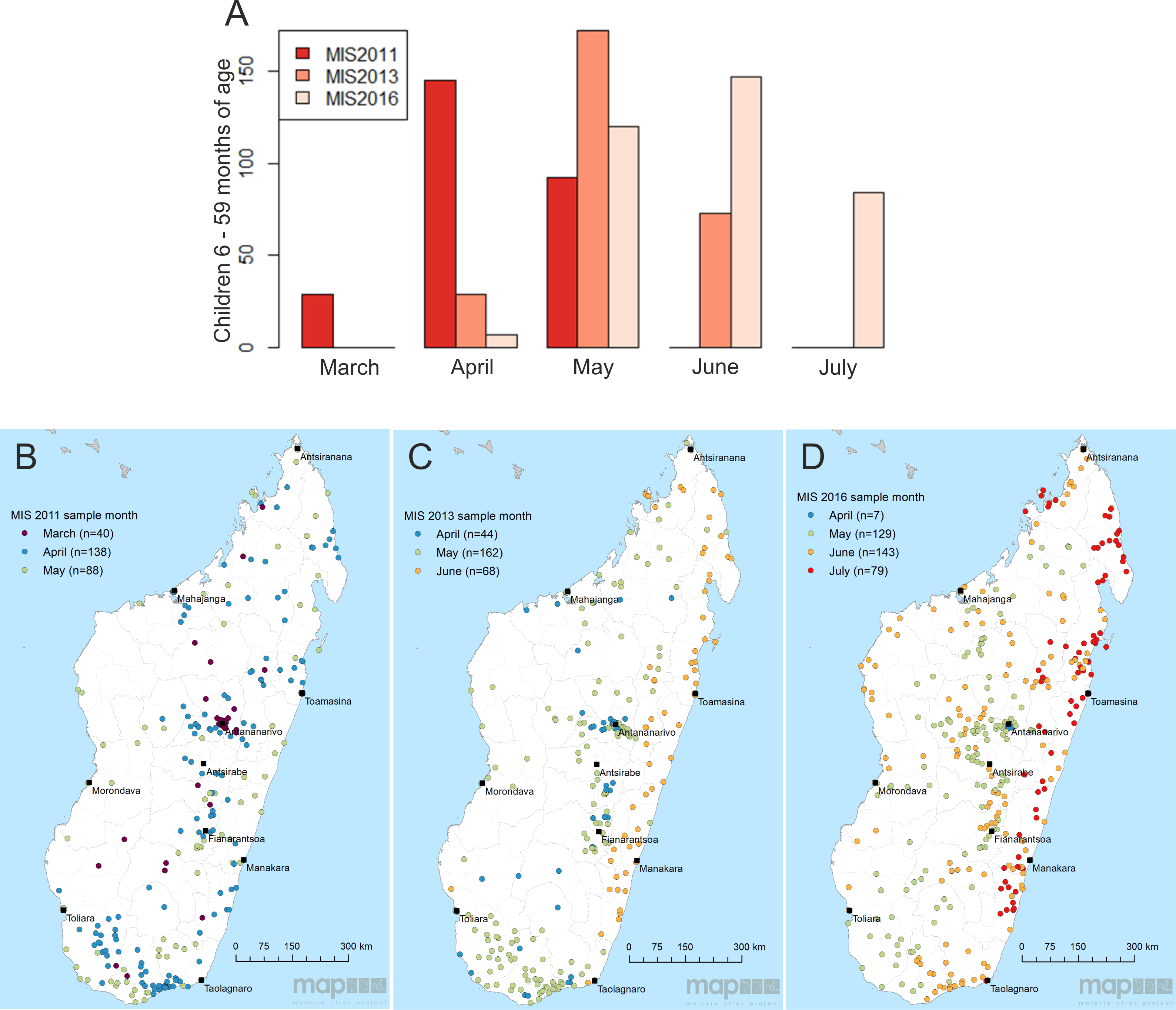

Supplement: Supplementary file 1 — Figure S1. Sample screening during the three MIS events in Madagascar, showing the progressive delay in the sampling time window. a Overall bar plots of sampling months. b–d Maps by sampling month by cluster location. (PNG 1353 kb) [file 12916_2018_1060_MOESM1_ESM.png]

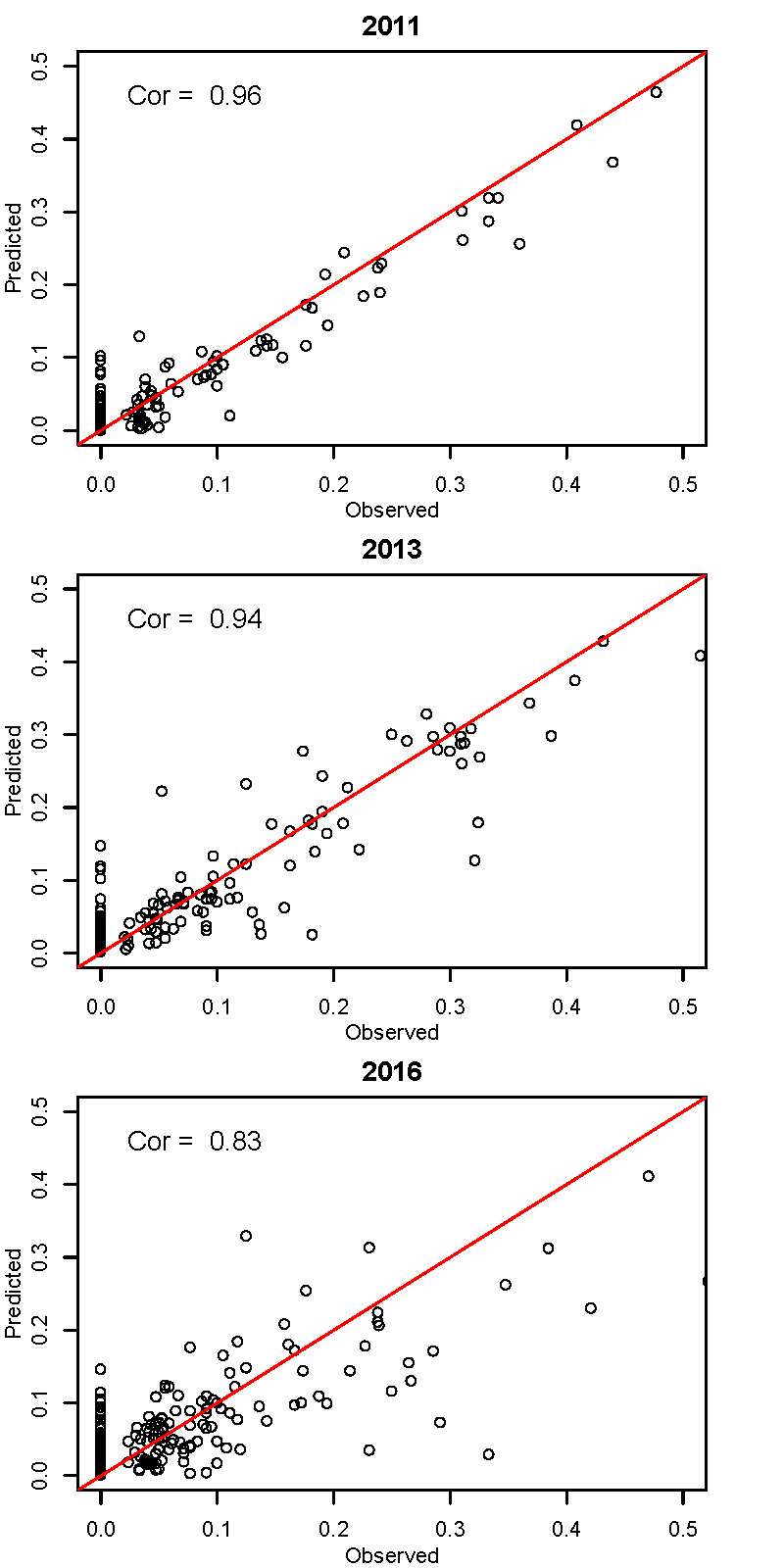

Supplement: Supplementary file 2 — Figure S2. Month-specific correlations between observed raw MIS prevalence values and the model predictions for each annual map. Pearson correlation coefficients are shown on each plot. (PNG 32 kb) [file 12916_2018_1060_MOESM2_ESM.png]

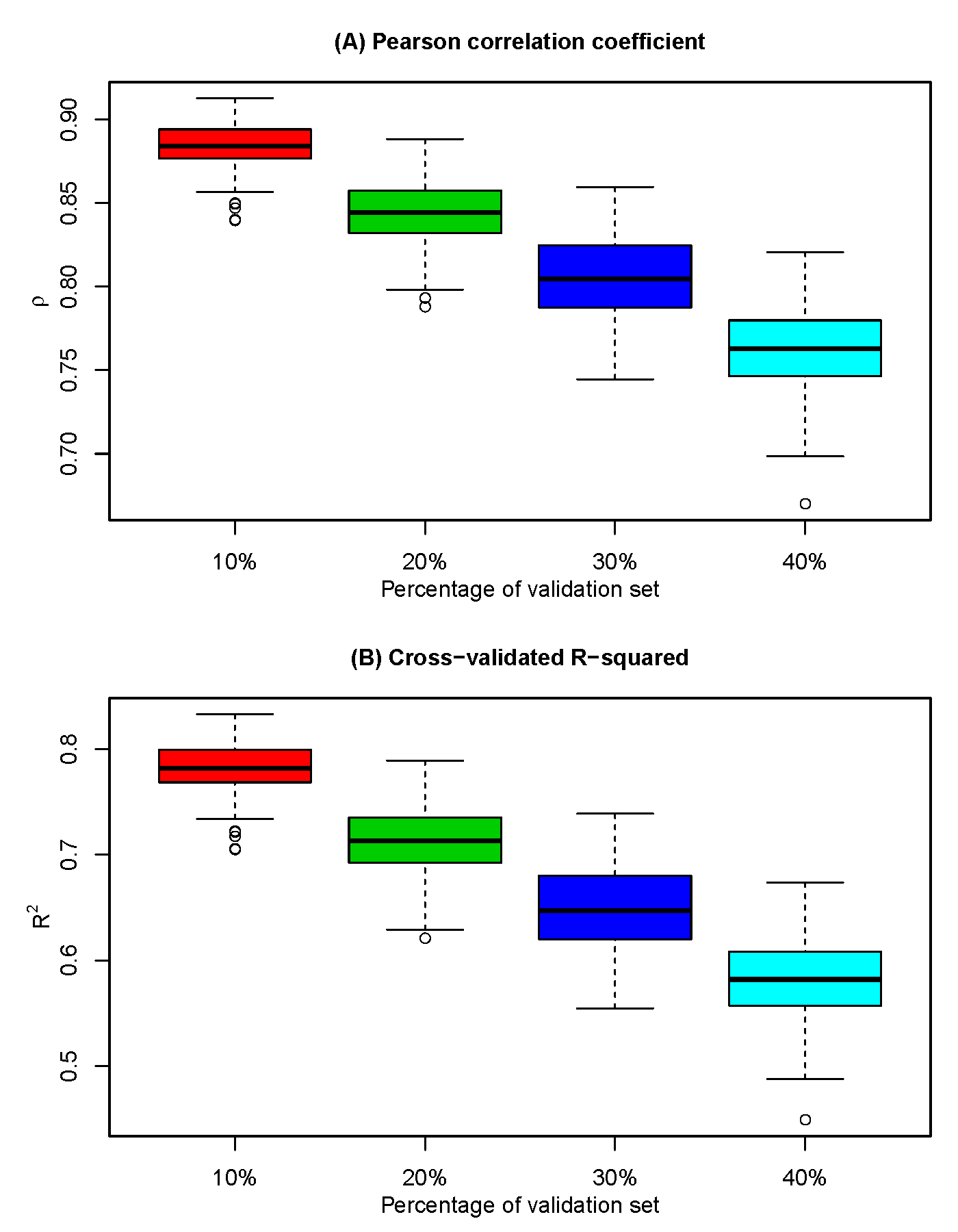

Supplement: Supplementary file 3 — Figure S3. Box plots of a cross-validated Pearson correlation coefficients and b cross-validated R2, based on 100 randomly sampled validation sets. (PNG 26 kb) [file 12916_2018_1060_MOESM3_ESM.png]

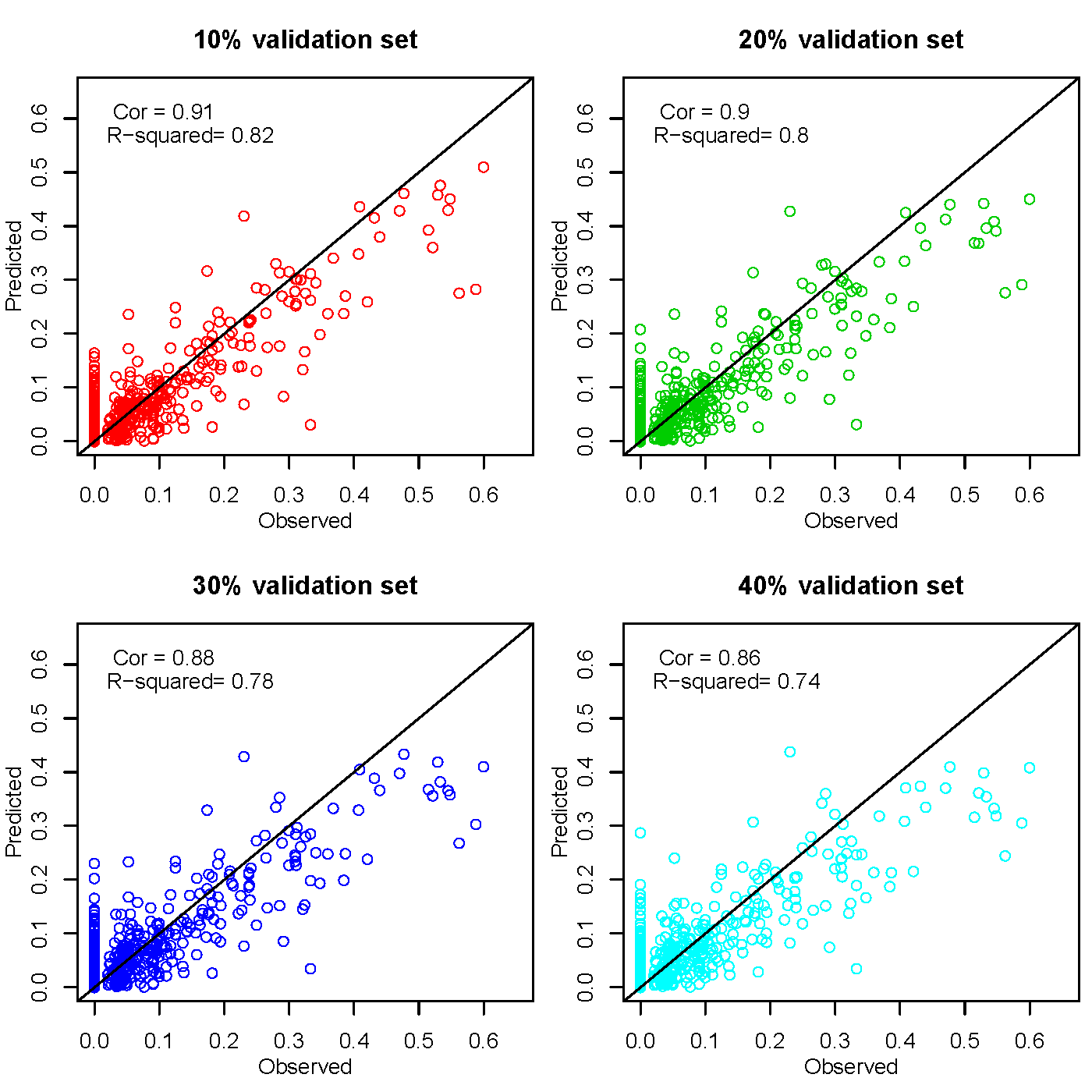

Supplement: Supplementary file 4 — Figure S4. Observed prevalence against predicted prevalence averaged across 100 randomly sampled validation sets. (PNG 63 kb) [file 12916_2018_1060_MOESM4_ESM.png]

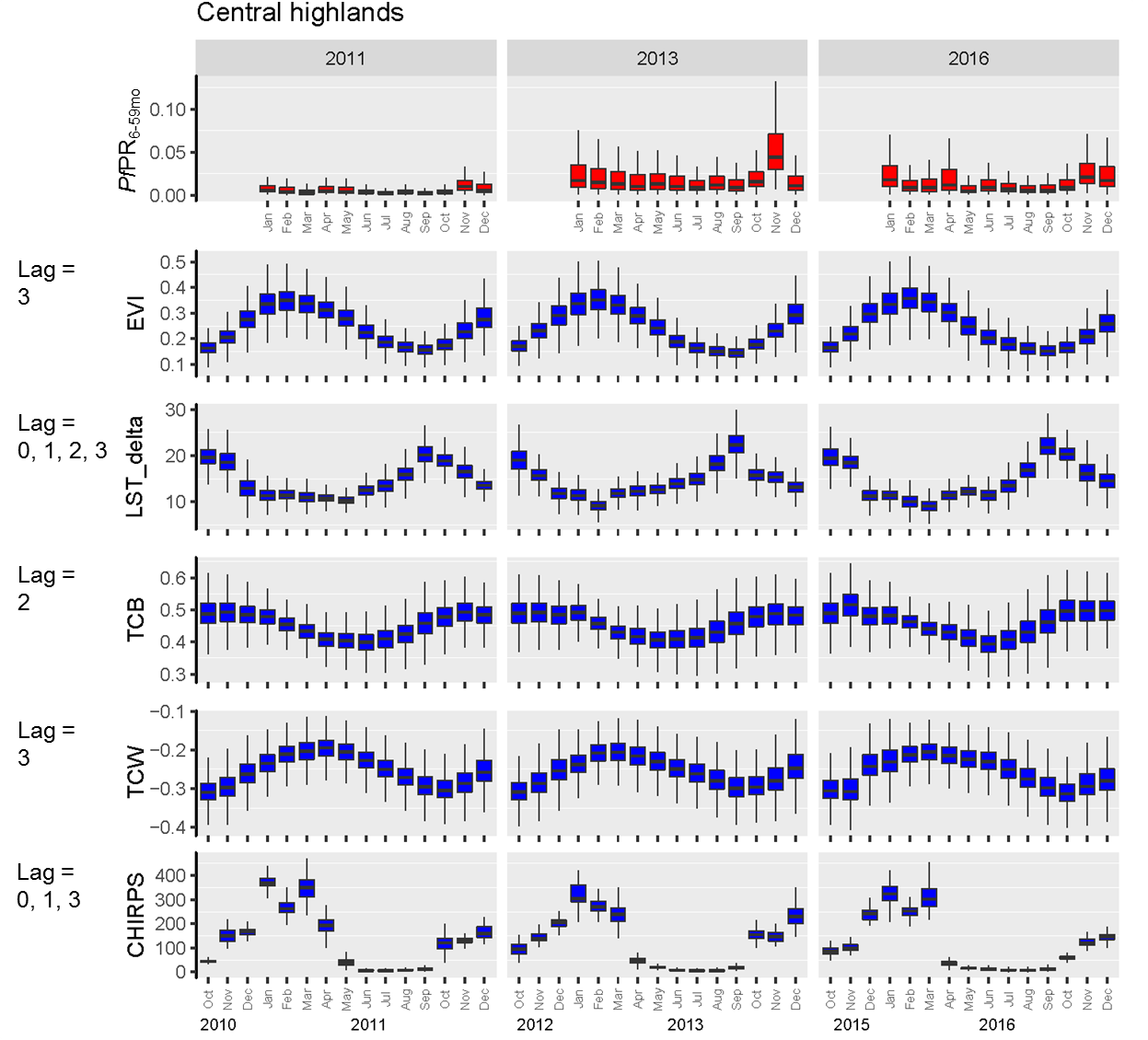

Supplement: Supplementary file 5 — Figure S5. Summary box plots of predicted monthly PfPR6–59mo by ecozone, plotted alongside temporally variable predictor values. The box plot rectangles indicate the first to third quartiles (interquartile range), with the median shown as the dark line inside the box. Vertical lines correspond to the minimum and maximum values. Specified lags indicate the time points that were selected by the model as explanatory variables of PfPR6–59mo. A time lag of 0 indicates that the covariate values in the concurrent month were predictive of PfPR6–59mo, while a time lag of 3 indicates that the covariate value 3 months prior to the prediction was predictive of PfPR6–59mo. (ZIP 1859 kb) [file 12916_2018_1060_MOESM5_ESM.zip › Kang_FigureS5HR2.png]

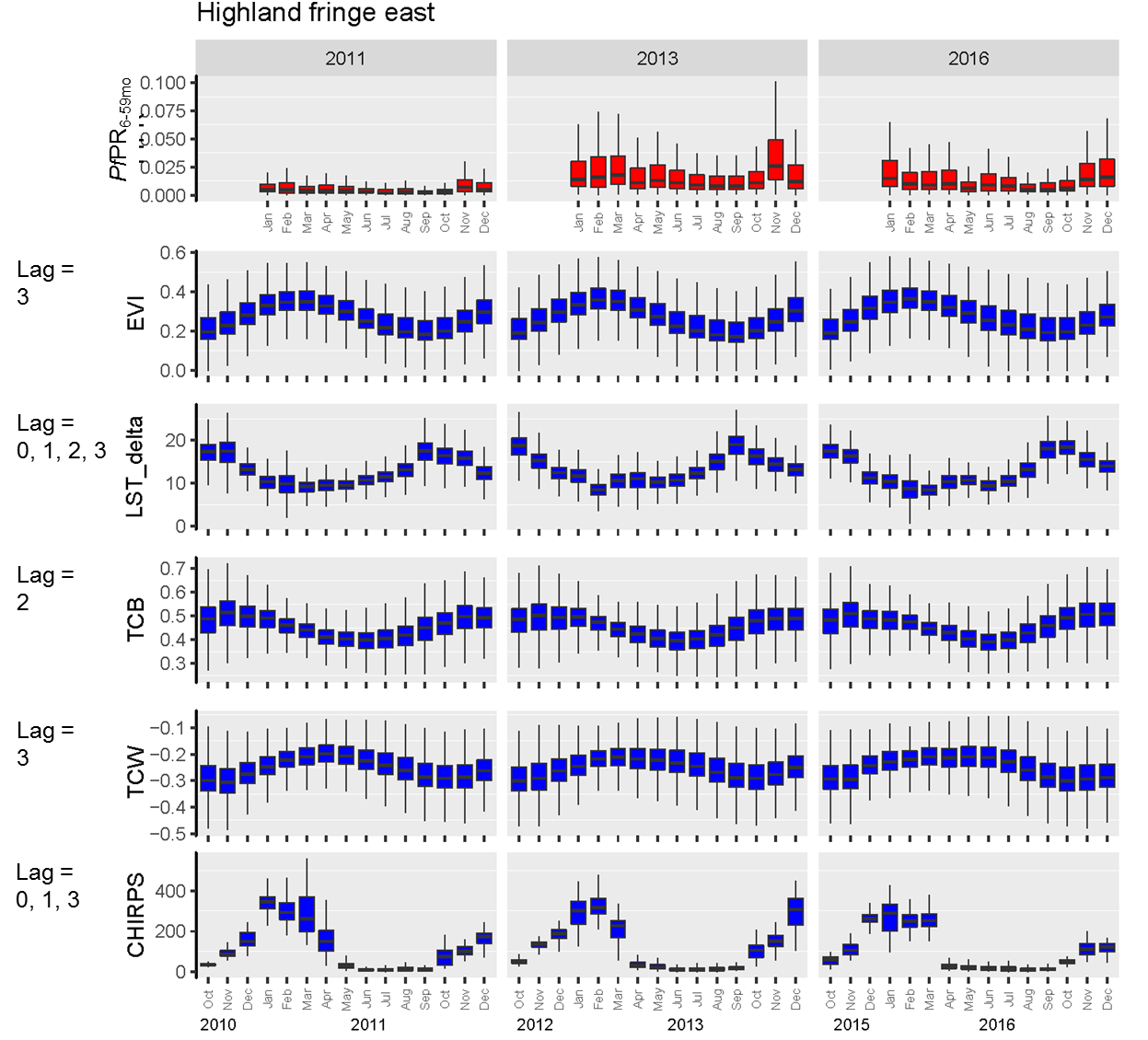

Supplement: Supplementary file 5 — Figure S5. Summary box plots of predicted monthly PfPR6–59mo by ecozone, plotted alongside temporally variable predictor values. The box plot rectangles indicate the first to third quartiles (interquartile range), with the median shown as the dark line inside the box. Vertical lines correspond to the minimum and maximum values. Specified lags indicate the time points that were selected by the model as explanatory variables of PfPR6–59mo. A time lag of 0 indicates that the covariate values in the concurrent month were predictive of PfPR6–59mo, while a time lag of 3 indicates that the covariate value 3 months prior to the prediction was predictive of PfPR6–59mo. (ZIP 1859 kb) [file 12916_2018_1060_MOESM5_ESM.zip › Kang_FigureS5FR2.png]

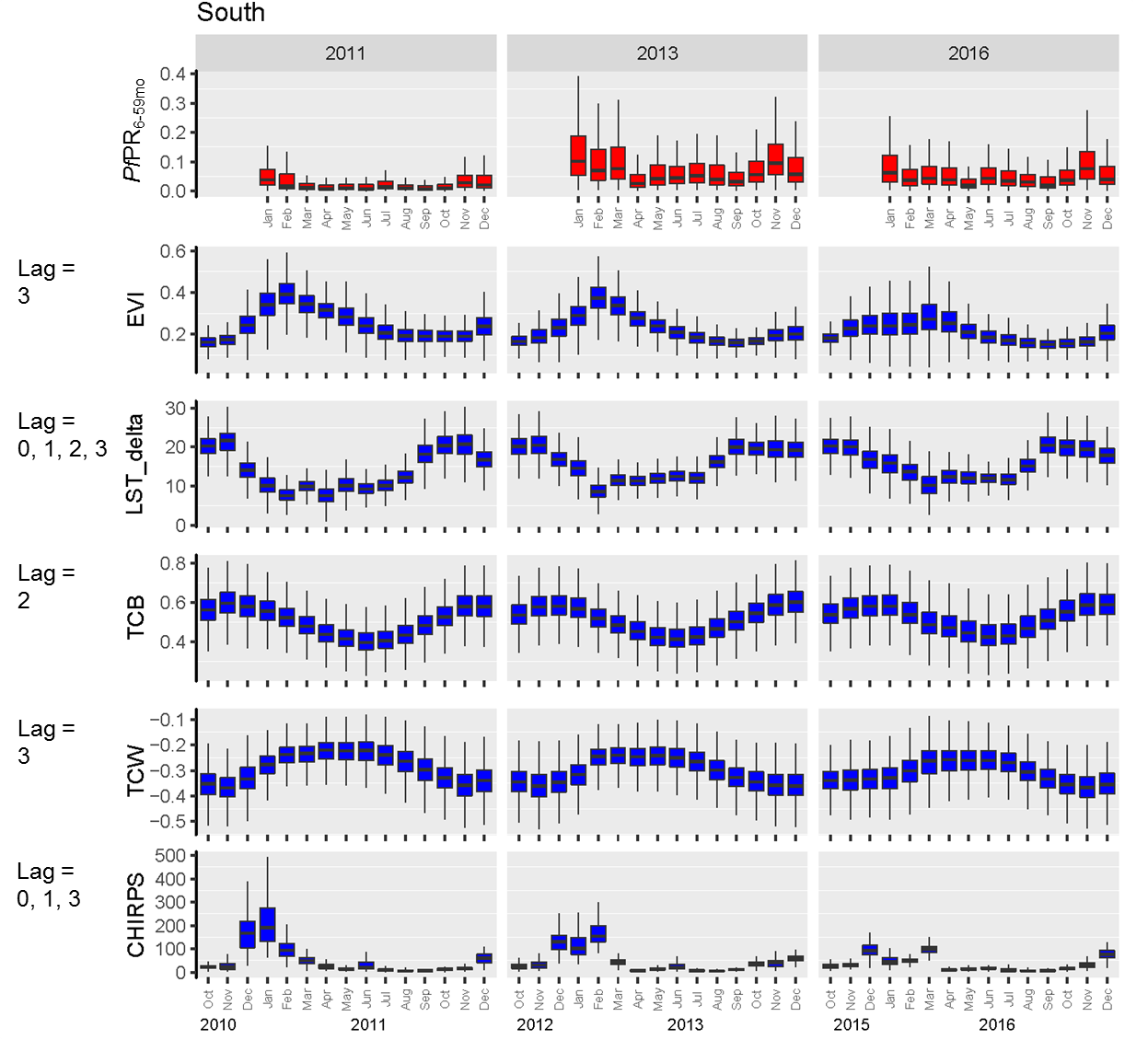

Supplement: Supplementary file 5 — Figure S5. Summary box plots of predicted monthly PfPR6–59mo by ecozone, plotted alongside temporally variable predictor values. The box plot rectangles indicate the first to third quartiles (interquartile range), with the median shown as the dark line inside the box. Vertical lines correspond to the minimum and maximum values. Specified lags indicate the time points that were selected by the model as explanatory variables of PfPR6–59mo. A time lag of 0 indicates that the covariate values in the concurrent month were predictive of PfPR6–59mo, while a time lag of 3 indicates that the covariate value 3 months prior to the prediction was predictive of PfPR6–59mo. (ZIP 1859 kb) [file 12916_2018_1060_MOESM5_ESM.zip › Kang_FigureS5GR2.png]

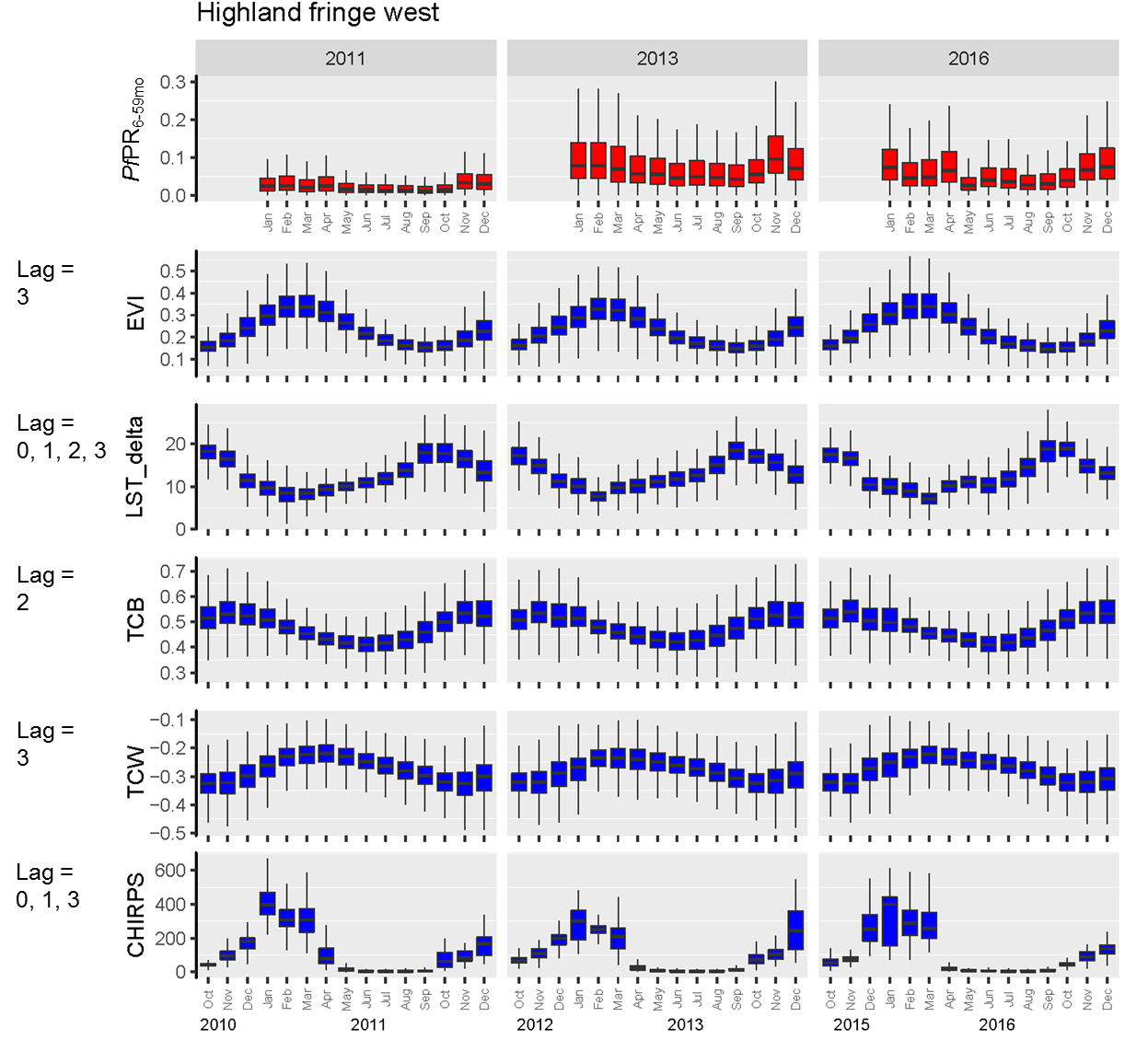

Supplement: Supplementary file 5 — Figure S5. Summary box plots of predicted monthly PfPR6–59mo by ecozone, plotted alongside temporally variable predictor values. The box plot rectangles indicate the first to third quartiles (interquartile range), with the median shown as the dark line inside the box. Vertical lines correspond to the minimum and maximum values. Specified lags indicate the time points that were selected by the model as explanatory variables of PfPR6–59mo. A time lag of 0 indicates that the covariate values in the concurrent month were predictive of PfPR6–59mo, while a time lag of 3 indicates that the covariate value 3 months prior to the prediction was predictive of PfPR6–59mo. (ZIP 1859 kb) [file 12916_2018_1060_MOESM5_ESM.zip › Kang_FigureS5ER2.png]

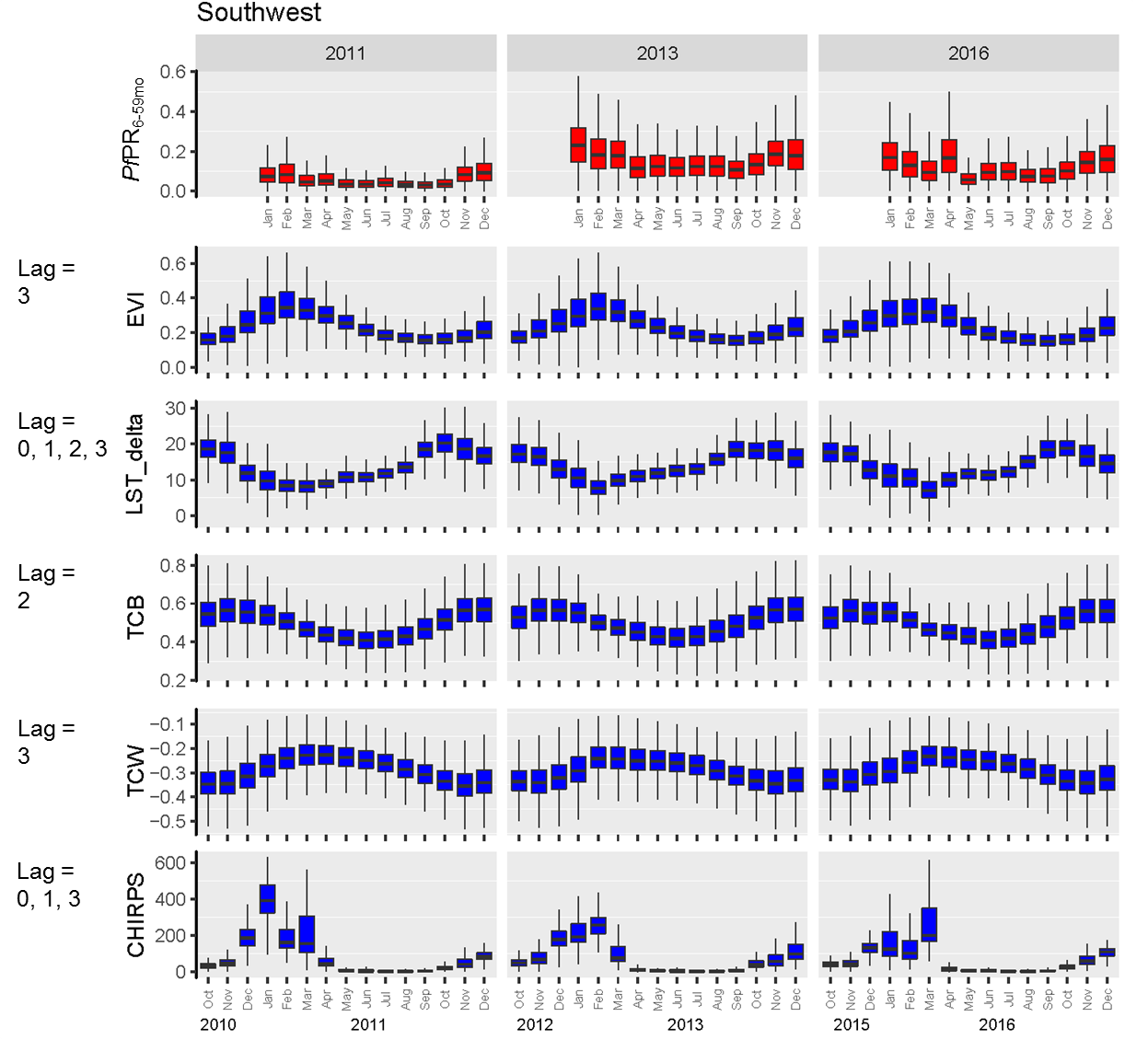

Supplement: Supplementary file 5 — Figure S5. Summary box plots of predicted monthly PfPR6–59mo by ecozone, plotted alongside temporally variable predictor values. The box plot rectangles indicate the first to third quartiles (interquartile range), with the median shown as the dark line inside the box. Vertical lines correspond to the minimum and maximum values. Specified lags indicate the time points that were selected by the model as explanatory variables of PfPR6–59mo. A time lag of 0 indicates that the covariate values in the concurrent month were predictive of PfPR6–59mo, while a time lag of 3 indicates that the covariate value 3 months prior to the prediction was predictive of PfPR6–59mo. (ZIP 1859 kb) [file 12916_2018_1060_MOESM5_ESM.zip › Kang_FigureS5CR2.png]

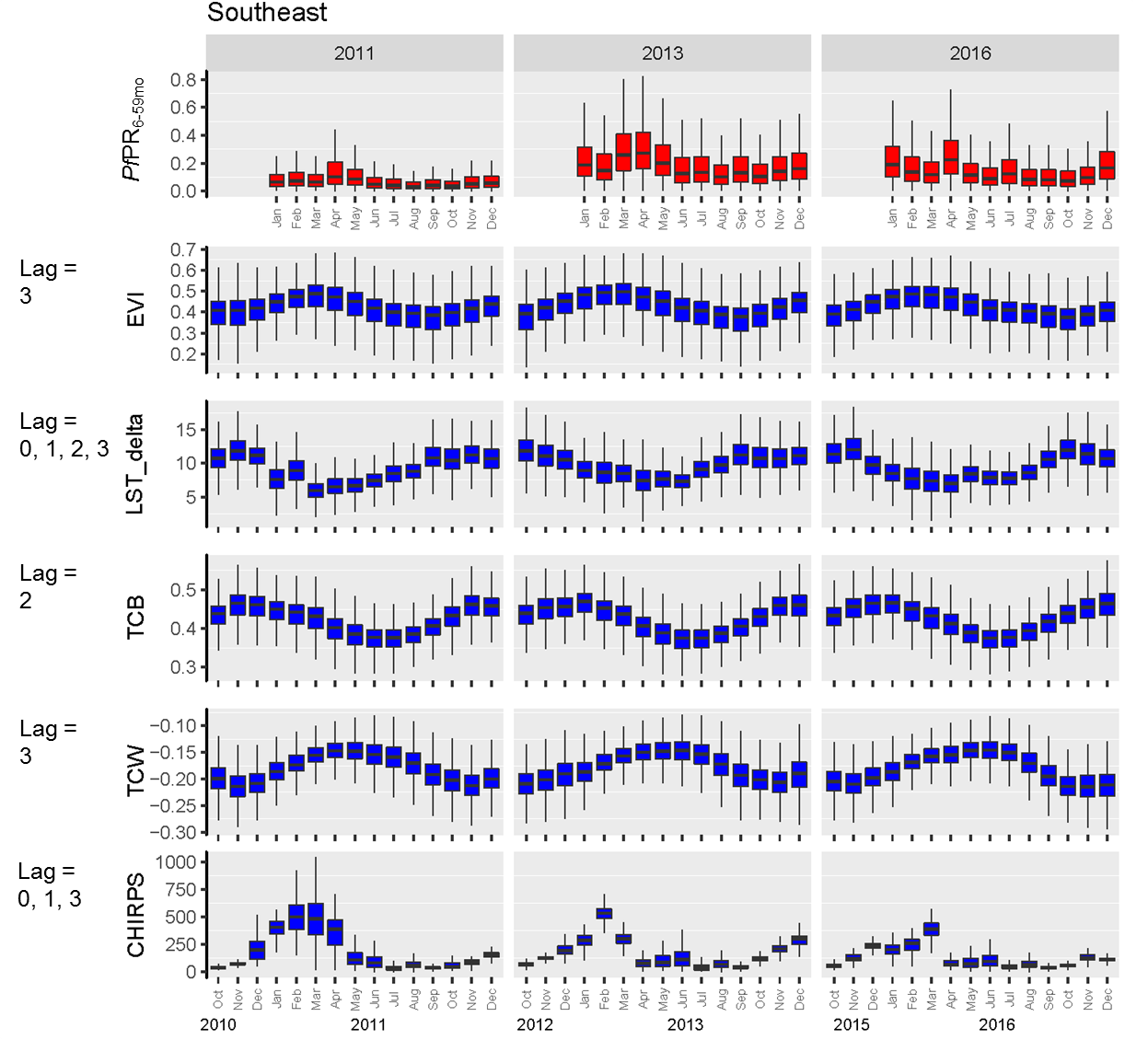

Supplement: Supplementary file 5 — Figure S5. Summary box plots of predicted monthly PfPR6–59mo by ecozone, plotted alongside temporally variable predictor values. The box plot rectangles indicate the first to third quartiles (interquartile range), with the median shown as the dark line inside the box. Vertical lines correspond to the minimum and maximum values. Specified lags indicate the time points that were selected by the model as explanatory variables of PfPR6–59mo. A time lag of 0 indicates that the covariate values in the concurrent month were predictive of PfPR6–59mo, while a time lag of 3 indicates that the covariate value 3 months prior to the prediction was predictive of PfPR6–59mo. (ZIP 1859 kb) [file 12916_2018_1060_MOESM5_ESM.zip › Kang_FigureS5DR2.png]

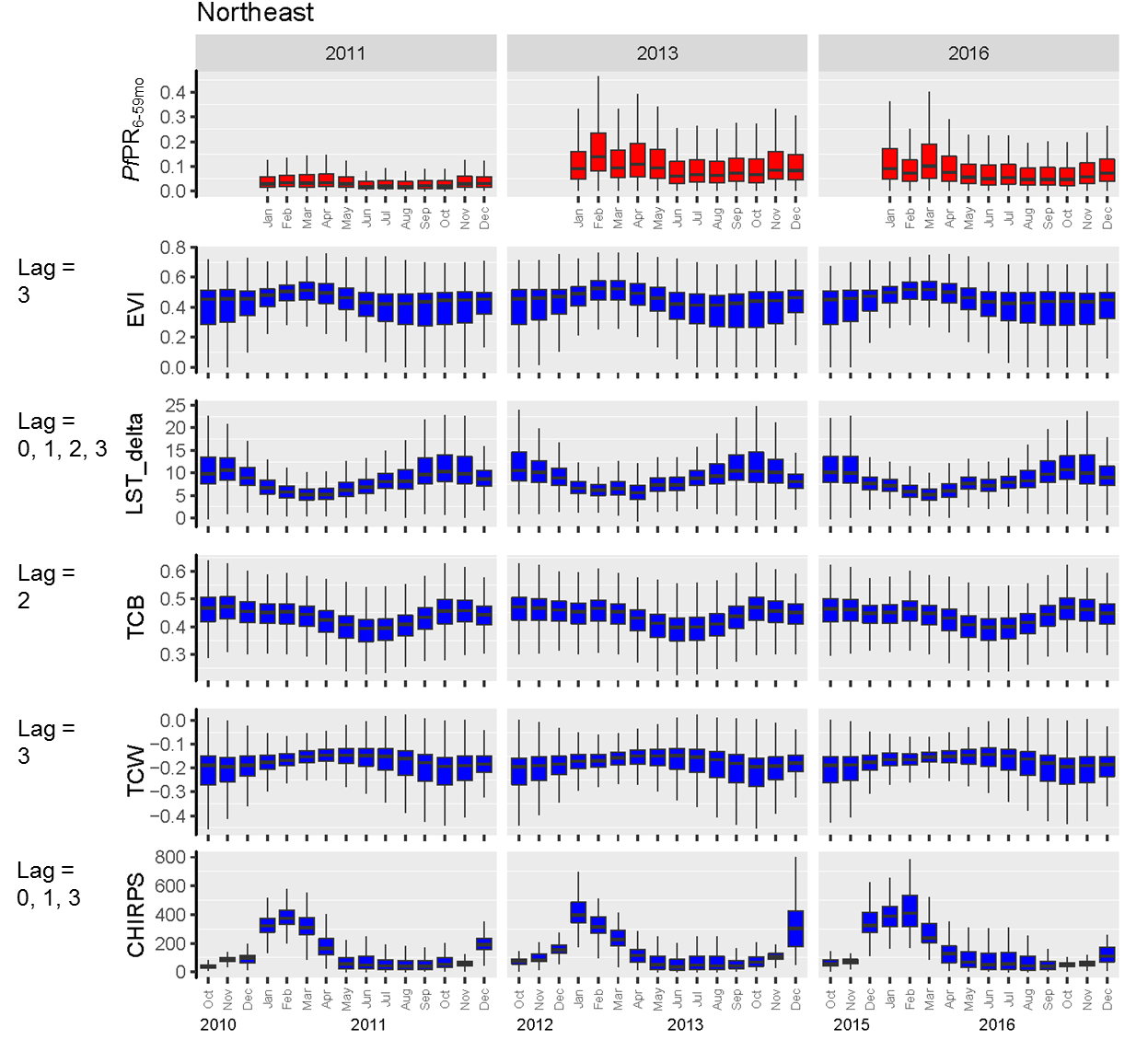

Supplement: Supplementary file 5 — Figure S5. Summary box plots of predicted monthly PfPR6–59mo by ecozone, plotted alongside temporally variable predictor values. The box plot rectangles indicate the first to third quartiles (interquartile range), with the median shown as the dark line inside the box. Vertical lines correspond to the minimum and maximum values. Specified lags indicate the time points that were selected by the model as explanatory variables of PfPR6–59mo. A time lag of 0 indicates that the covariate values in the concurrent month were predictive of PfPR6–59mo, while a time lag of 3 indicates that the covariate value 3 months prior to the prediction was predictive of PfPR6–59mo. (ZIP 1859 kb) [file 12916_2018_1060_MOESM5_ESM.zip › Kang_FigureS5BR2.png]

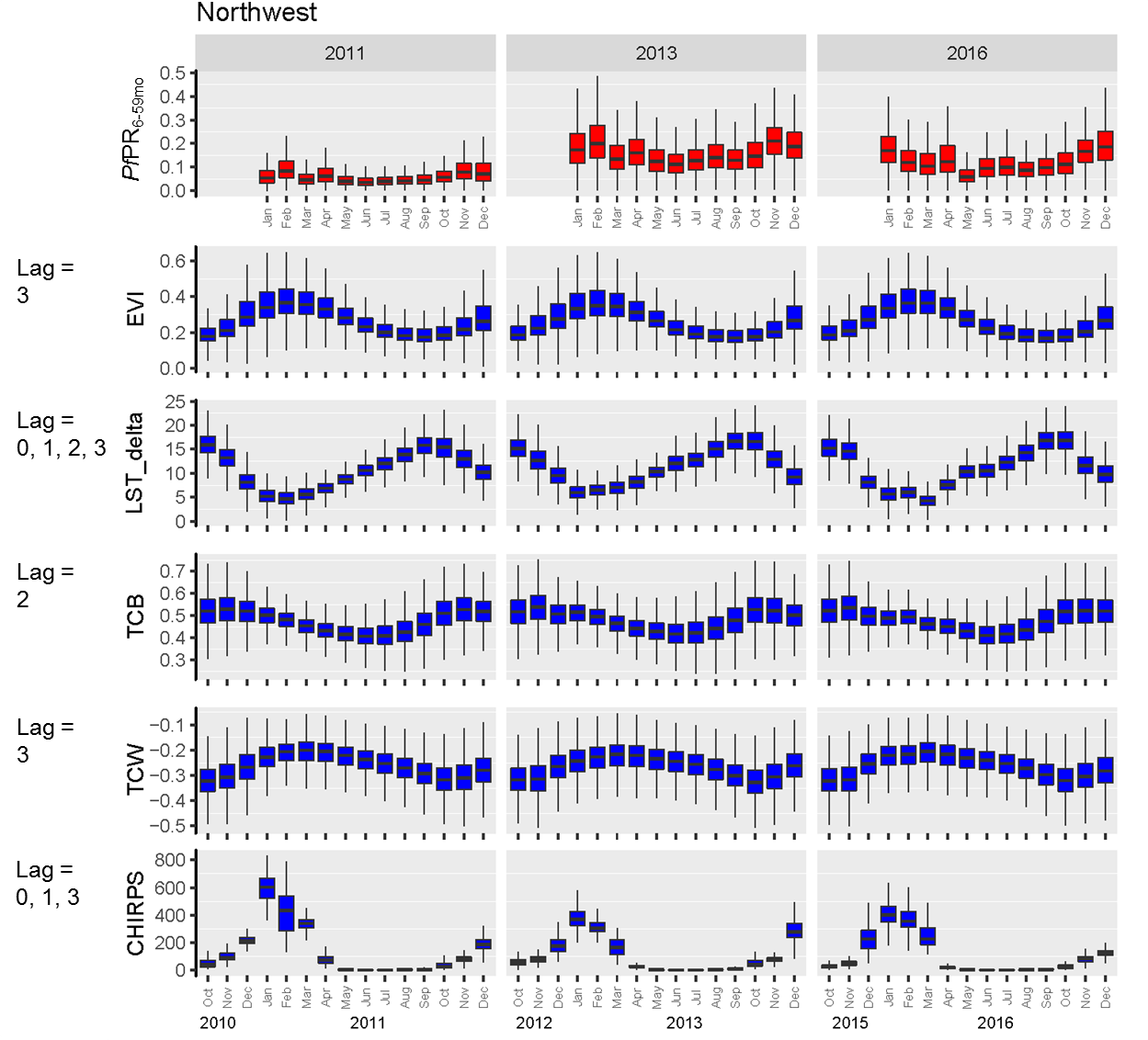

Supplement: Supplementary file 5 — Figure S5. Summary box plots of predicted monthly PfPR6–59mo by ecozone, plotted alongside temporally variable predictor values. The box plot rectangles indicate the first to third quartiles (interquartile range), with the median shown as the dark line inside the box. Vertical lines correspond to the minimum and maximum values. Specified lags indicate the time points that were selected by the model as explanatory variables of PfPR6–59mo. A time lag of 0 indicates that the covariate values in the concurrent month were predictive of PfPR6–59mo, while a time lag of 3 indicates that the covariate value 3 months prior to the prediction was predictive of PfPR6–59mo. (ZIP 1859 kb) [file 12916_2018_1060_MOESM5_ESM.zip › Kang_FigureS5AR2.png]

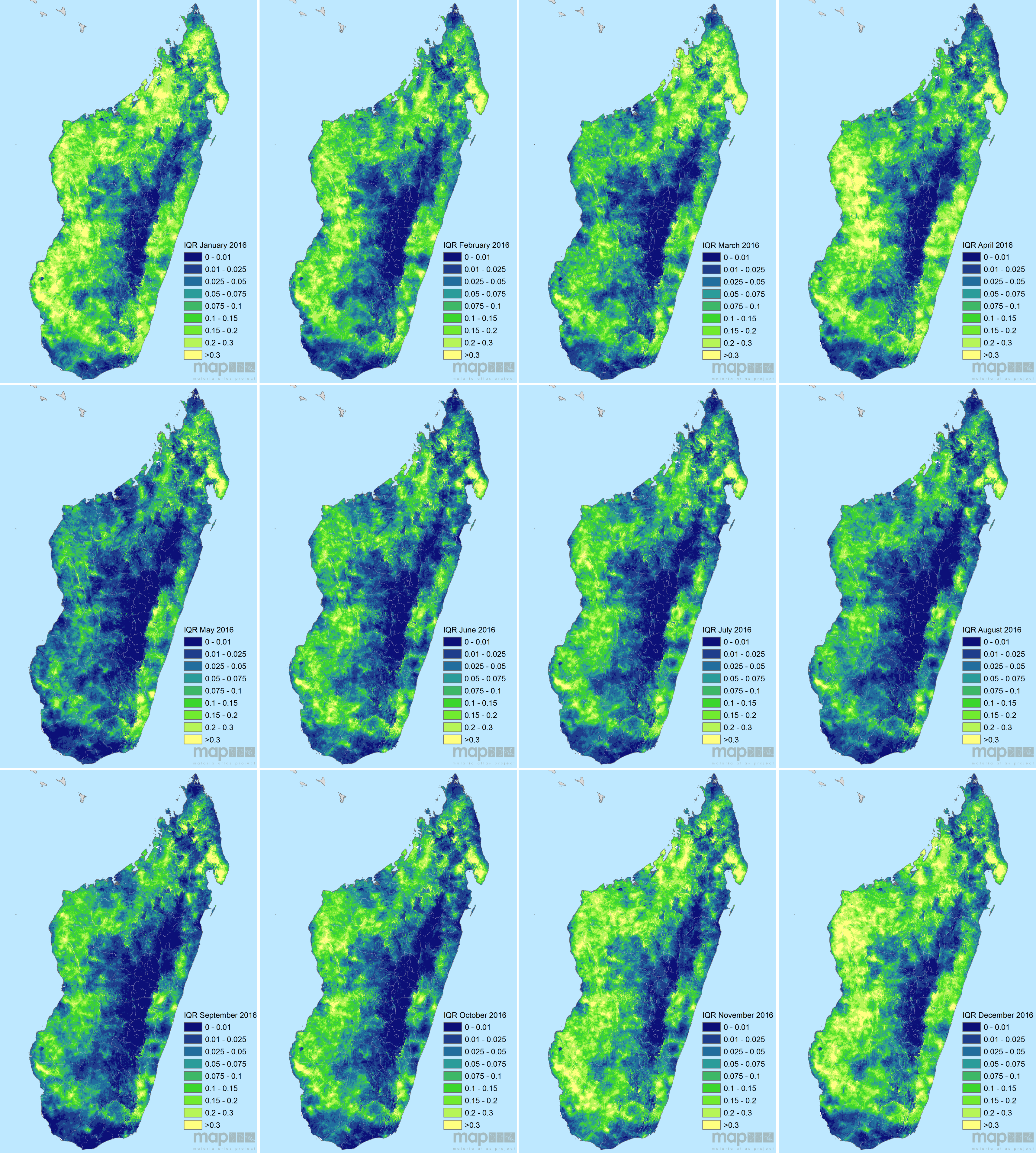

Supplement: Supplementary file 6 — Figure S6. Predicted monthly mean PfPR6–59mo maps for a 2011, c 2013 and e 2016, with associated uncertainty (interquartile range) for b 2011, d 2013 and f 2016. (ZIP 48310 kb) [file 12916_2018_1060_MOESM6_ESM.zip › Kang_FigureS6FR2.png]

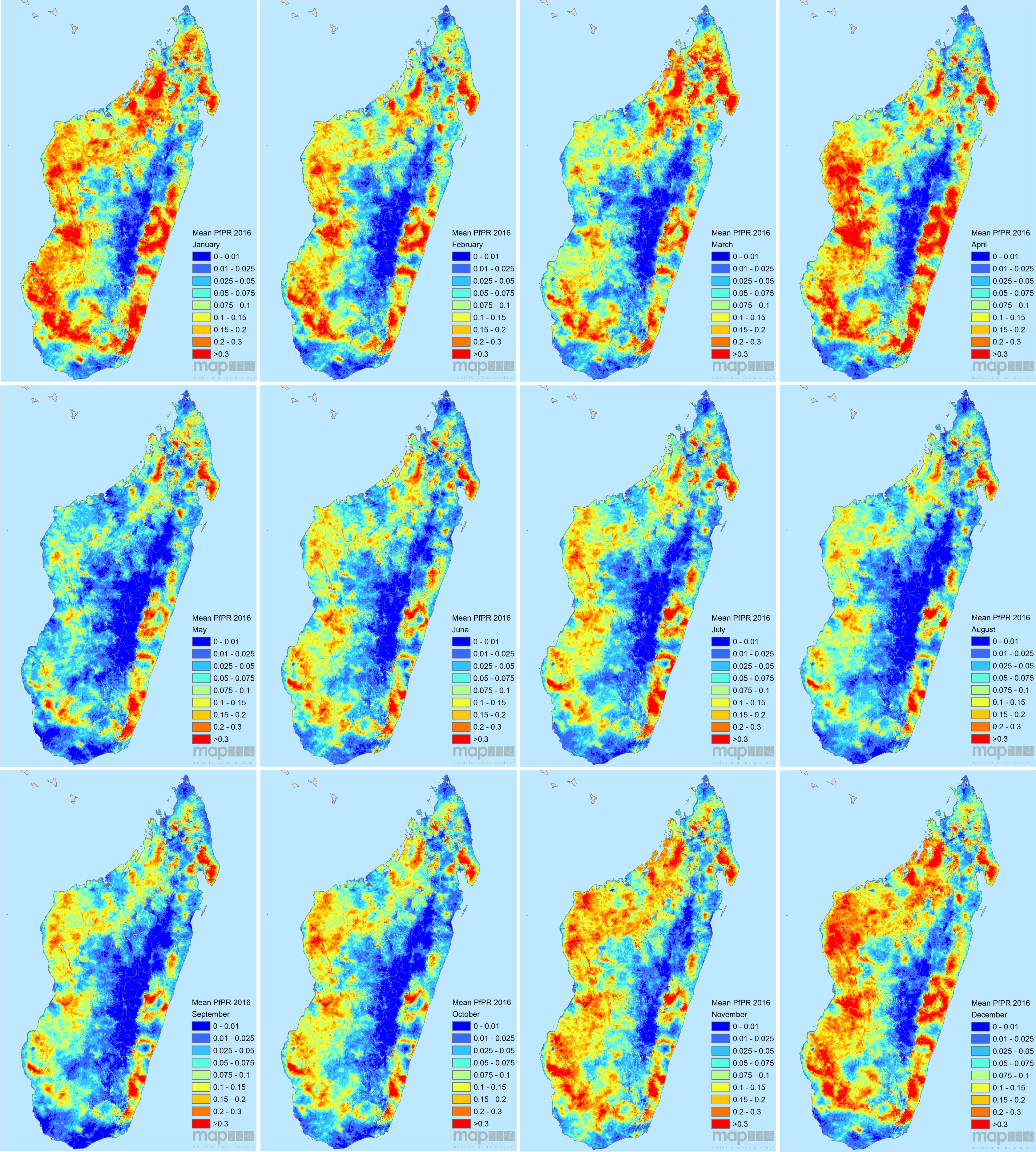

Supplement: Supplementary file 6 — Figure S6. Predicted monthly mean PfPR6–59mo maps for a 2011, c 2013 and e 2016, with associated uncertainty (interquartile range) for b 2011, d 2013 and f 2016. (ZIP 48310 kb) [file 12916_2018_1060_MOESM6_ESM.zip › Kang_FigureS6ER2.jpg]

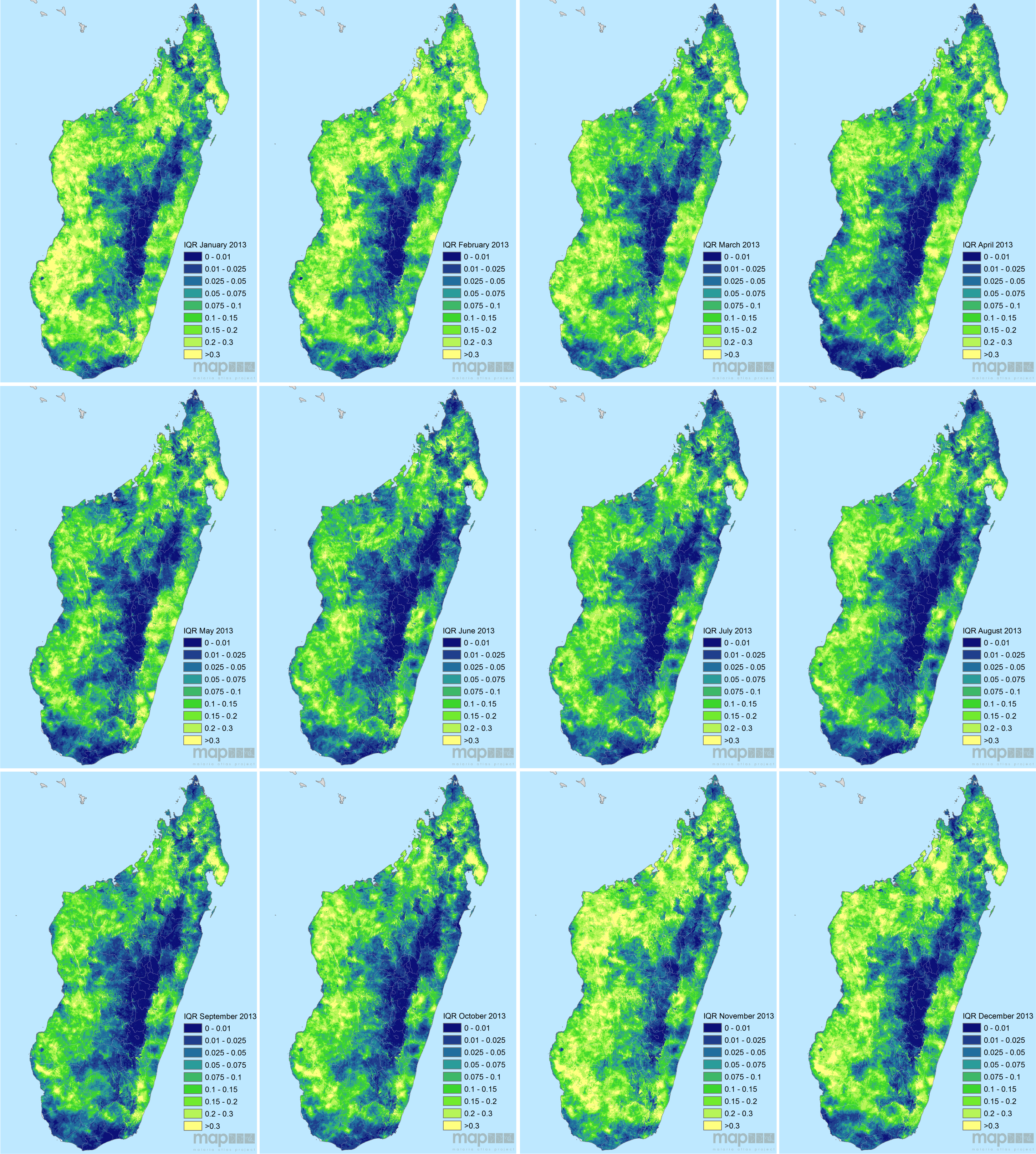

Supplement: Supplementary file 6 — Figure S6. Predicted monthly mean PfPR6–59mo maps for a 2011, c 2013 and e 2016, with associated uncertainty (interquartile range) for b 2011, d 2013 and f 2016. (ZIP 48310 kb) [file 12916_2018_1060_MOESM6_ESM.zip › Kang_FigureS6DR2.png]

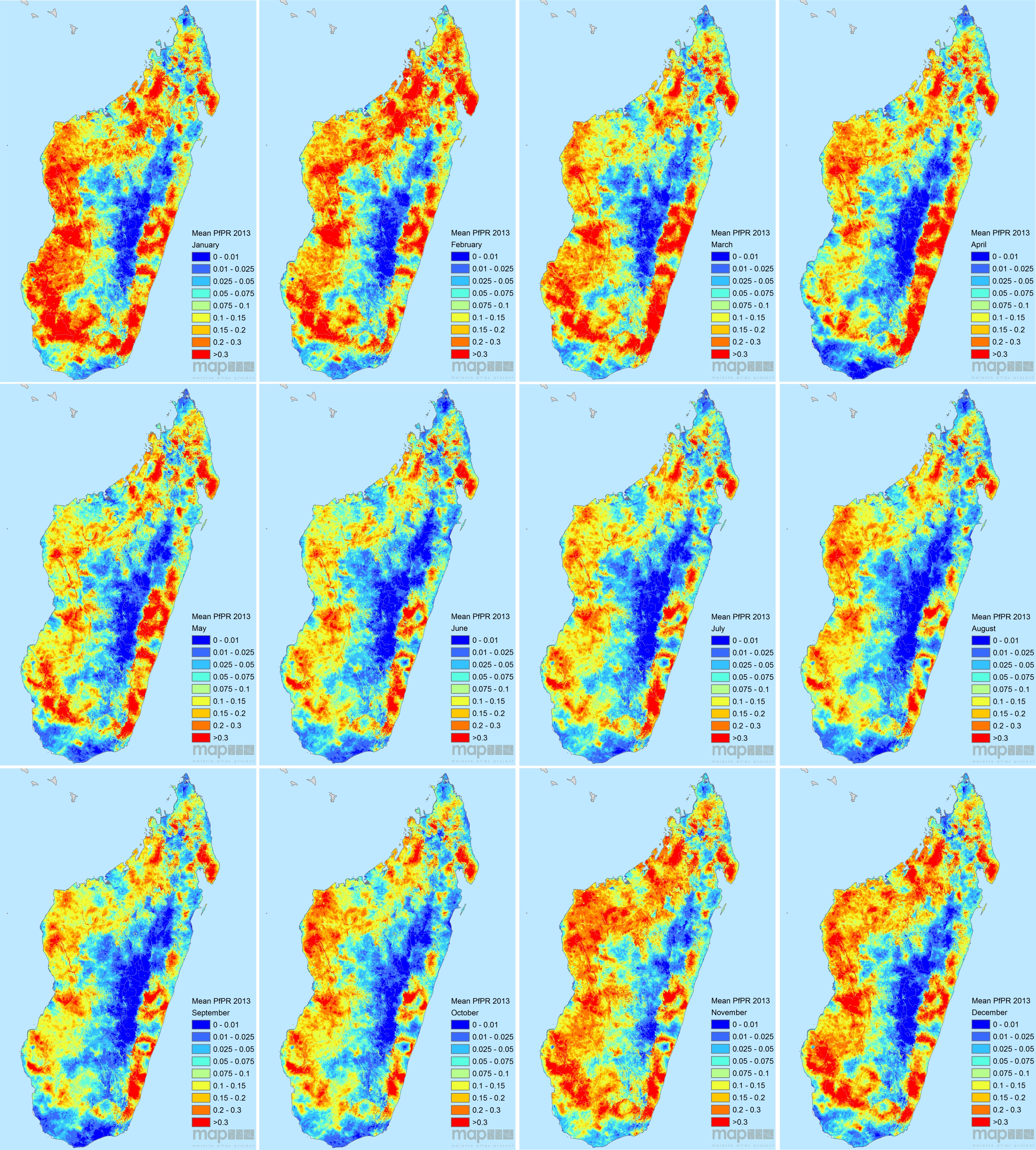

Supplement: Supplementary file 6 — Figure S6. Predicted monthly mean PfPR6–59mo maps for a 2011, c 2013 and e 2016, with associated uncertainty (interquartile range) for b 2011, d 2013 and f 2016. (ZIP 48310 kb) [file 12916_2018_1060_MOESM6_ESM.zip › Kang_FigureS6CR2.jpg]

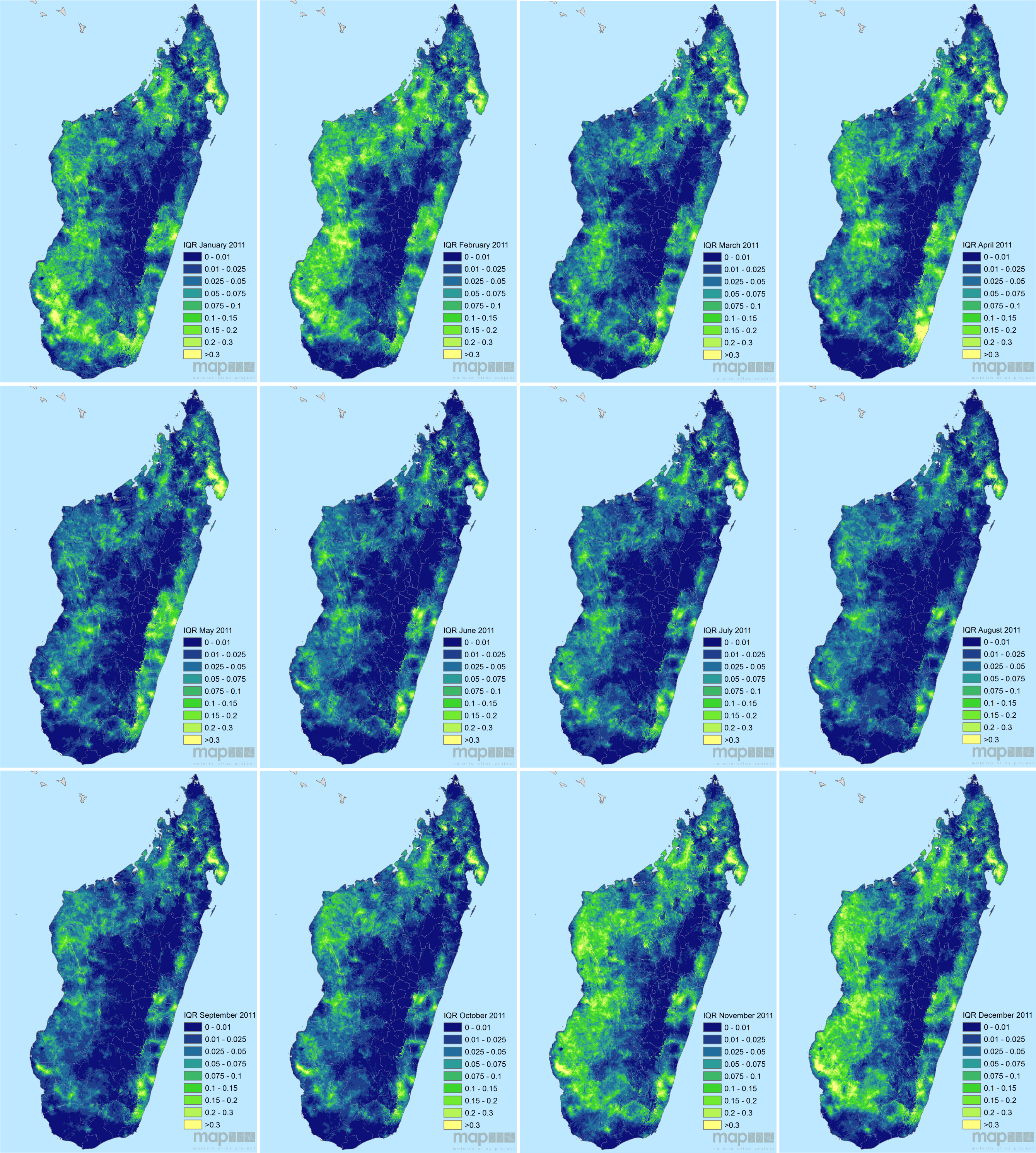

Supplement: Supplementary file 6 — Figure S6. Predicted monthly mean PfPR6–59mo maps for a 2011, c 2013 and e 2016, with associated uncertainty (interquartile range) for b 2011, d 2013 and f 2016. (ZIP 48310 kb) [file 12916_2018_1060_MOESM6_ESM.zip › Kang_FigureS6BR2.png]

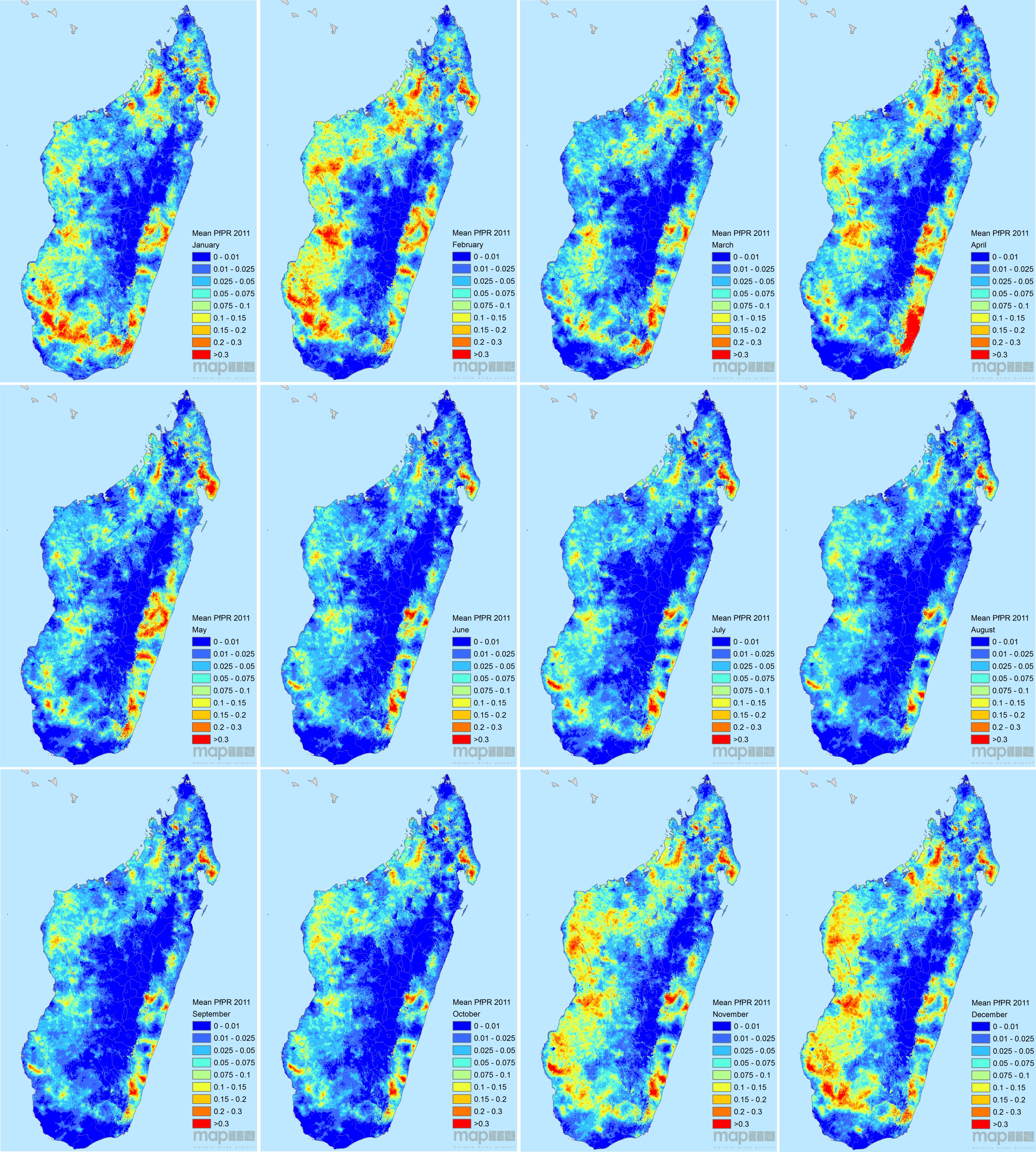

Supplement: Supplementary file 6 — Figure S6. Predicted monthly mean PfPR6–59mo maps for a 2011, c 2013 and e 2016, with associated uncertainty (interquartile range) for b 2011, d 2013 and f 2016. (ZIP 48310 kb) [file 12916_2018_1060_MOESM6_ESM.zip › Kang_FigureS6AR2.jpg]

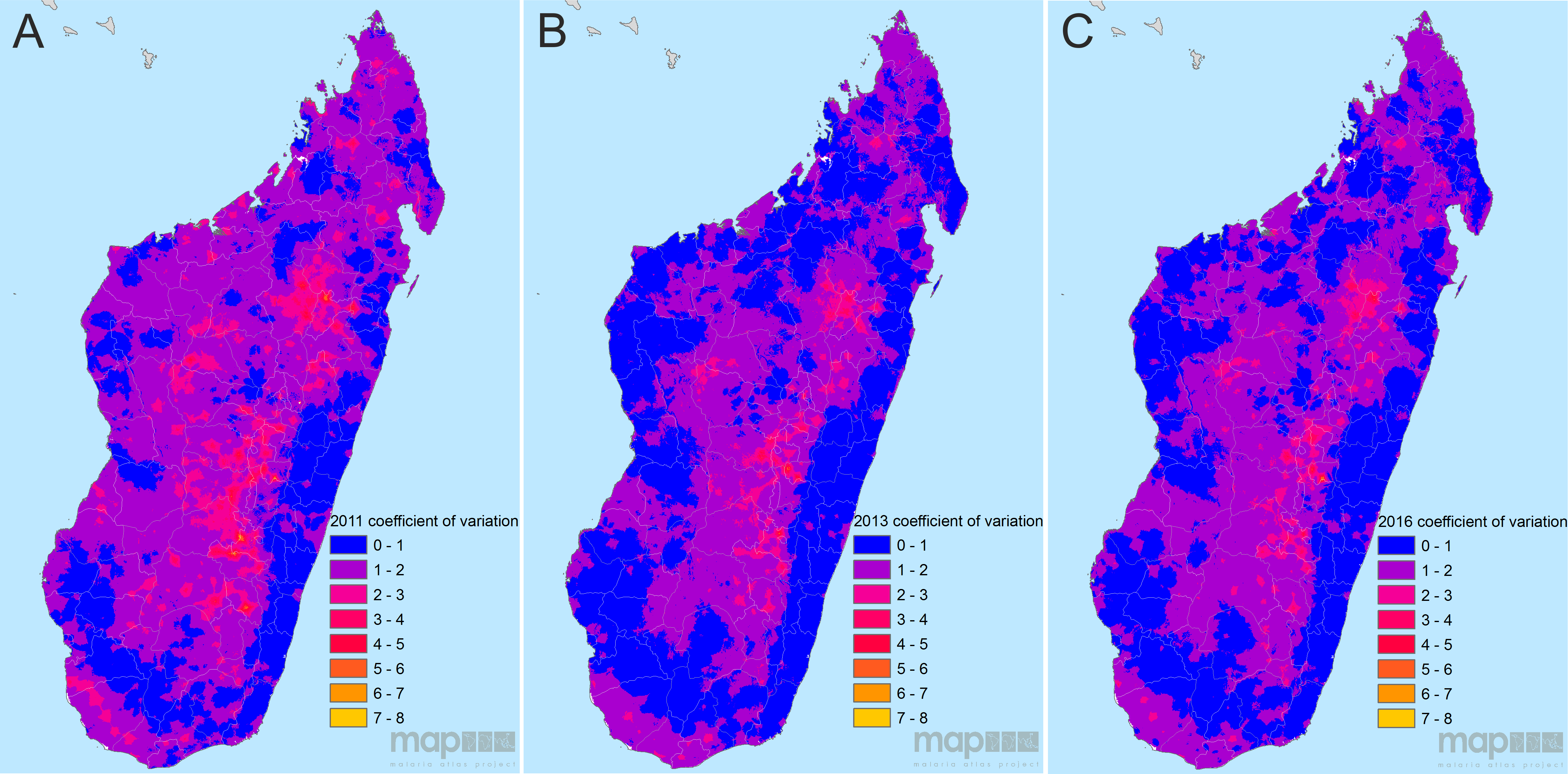

Supplement: Supplementary file 7 — Figure S7. Maps of the coefficient of variation in the annual mean maps (standard deviation/annual mean) showing the relative confidence in the predictions across the country for a 2011, b 2013 and c 2016. (PNG 535 kb) [file 12916_2018_1060_MOESM7_ESM.png]
